# A Nanovaccine Based on Adjuvant Peptide FK‐13 and l‐Phenylalanine Poly(ester amide) Enhances CD8^+^ T Cell‐Mediated Antitumor Immunity

**DOI:** 10.1002/advs.202300418

**Published:** 2023-05-10

**Authors:** Chunyuan Xie, Xinru You, Hongxia Zhang, Jiahui Li, Liying Wang, Yongxiang Liu, Zining Wang, Ruhui Yao, Tong Tong, Mengyun Li, Xiaojuan Wang, Lei Cui, Huanling Zhang, Hui Guo, Chunwei Li, Jun Wu, Xiaojun Xia

**Affiliations:** ^1^ State Key Laboratory of Oncology in South China and Collaborative Innovation Center for Cancer Medicine Sun Yat‐sen University Cancer Center 651 Dongfeng East Road Guangzhou 510060 China; ^2^ Center for Nanomedicine and Department of Anesthesiology Brigham and Women's Hospital Harvard Medical School Boston MA 02115 USA; ^3^ School of Food Science and Technology National Engineering Research Center of Seafood Dalian Polytechnic University Dalian 116024 China; ^4^ School of Biomedical Engineering Sun Yat‐sen University 66 Gongchang Road Shenzhen 518107 China; ^5^ State Key Laboratory of Biocontrol School of Life Science Sun Yat‐sen University 135 Xingang West Road Guangzhou 510275 China; ^6^ Bioscience and Biomedical Engineering Thrust The Hong Kong University of Science and Technology (Guangzhou) Nansha Guangzhou 511400 China; ^7^ Division of Life Science The Hong Kong University of Science and Technology Hong Kong SAR 999077 China

**Keywords:** adjuvant, FK‐13, immunotherapy, Phe‐PEA, tumor nanovaccine

## Abstract

Cancer vaccines have shown promise as effective means of antitumor immunotherapy by inducing tumor antigen‐specific T cell immunity. In this study, a novel peptide‐based tumor nanovaccine that boosts antigen presentation and elicits effective antitumor immunity is developed. The adjuvant characteristics of an antimicrobial peptide‐derived core peptide, FK‐13, are investigated and used it to generate a fusion peptide named FK‐33 with tumor antigen epitopes. l‐phenylalanine‐based poly(ester amide) (Phe‐PEA), 8p4, is also identified as a competent delivery vehicle for the fusion peptide FK‐33. Notably, the vaccination of 8p4 + FK‐33 nanoparticles (8FNs) in vivo induces dendritic cell activation in the lymph nodes and elicits robust tumor antigen‐specific CD8^+^ T cell response. The nanovaccine 8FNs demonstrate significant therapeutic and prophylactic efficacy against in situ tumor growth, effectively inhibit tumor metastasis, and significantly prolong the survival of tumor‐bearing mice. Moreover, 8FNs can incorporate different tumor antigens and exhibit a synergistic therapeutic effect with antiprogrammed cell death protein 1 (PD‐1) therapy. In summary, 8FNs represent a promising platform for personalized cancer vaccines and may serve as a potential combinational modality to improve current immunotherapy.

## Graphical abstract

A nanovaccine is designed using adjuvant peptide FK‐13 fused with antigen peptide, and l‐phenylalanine‐based poly(ester amide) polymers 8p4 as an efficient nanocarrier. The prepared nanovaccines named 8FNs can reverse the immunosuppressive tumor microenvironment, induce robust antitumor CD8^+^ T cell responses, inhibit tumor growth and metastasis, and show a synergistic therapeutic effect with anti‐PD‐1 therapy.

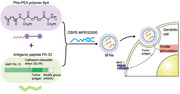

## 1 Introduction

Tumor immunotherapy is a promising strategy for treating malignancies by reactivating the immune system's cytotoxic potential, particularly tumor‐specific cytotoxic T cells.^[^ ^1^ ^]^ Although modern immunotherapies, such as adoptive T cell therapy and immune checkpoint blockade, which targets cytotoxic T lymphocyte antigen‐4, PD‐1 or its ligand (PD‐L1), have revolutionized the treatment of malignancies, a significant portion of patients still do not benefit from existing therapeutic options.^[^ ^2^ ^]^ Limitations, such as unreliable immune response rates, toxic side effects, and tumor‐intrinsic resistance to immunity, have prompted research for novel immunotherapy and appropriate combination therapies to enhance existing clinical treatment strategies.^[^ ^1^, ^2^, ^3^ ^]^ Unlike the limited clinical efficacy of past cancer vaccines, nanotechnology‐based tumor vaccines and tumor‐specific neoantigens hold promise as next‐generation immunotherapy, enabling patients to develop durable, specific, and effective anti‐tumor responses.^[^ ^4^ ^]^ The principle of tumor nanovaccines involves delivering tumor antigens by nanocarriers into antigen‐presenting cells (APCs), particularly dendritic cells (DCs), which then present major histocompatibility complex (MHC)‐bound epitopes to T cells to initiate a unique tumor‐killing process.^[^ ^5^ ^]^ For example, a soft mesoporous organosilica‐based nanovaccine could effectively promote DCs maturation and antigen cross‐presentation, resulting in robust antigen‐specific T‐cell responses.^[^ ^6^ ^]^ Compared to traditional vaccines, such as whole cell‐based vaccines and epitope‐based subunit vaccines, antigen peptide‐based vaccines are safer and can provoke more robust immune responses, particularly with the help of adjuvants.^[^ ^7^ ^]^ Peptide‐based tumor vaccines have been extensively studied in preclinical experiments and clinical trials due to their advantages, including good safety profiles, ease of manufacturing, and stable quality control.^[^ ^8^ ^]^ Despite the advantages of peptide‐based tumor vaccines, critical challenges still remain, such as identifying tumor‐specific T‐cell epitopes and overcoming the weak immunogenicity of peptide antigens, to achieve better therapeutic effects for peptide‐based cancer vaccines.^[^ ^9^ ^]^

With the rapid advances in sequencing and bioinformatics‐based neoantigen identification approaches, the significant challenge for effective peptide‐based cancer vaccines is how to efficiently deliver of candidate neoantigens into APCs and elicit robust antitumor immune responses in vivo.^[^ ^5^ ^]^ Another vital design consideration for peptide‐based vaccines is that neoepitopes of individual patients are distinctive, and tumor vaccination regimens usually consist of multiple priming and boost administration with profiles of peptide antigens, which requires an efficient delivery system.^[^ ^10^ ^]^ Currently, a variety of materials, such as metallic nanoparticles, modified carbon nanomaterials, silica nanoparticles, liposomes, and polymers, have been used as antigen delivery systems to promote the generation of tumor antigen‐specific T cells.^[^ ^11^ ^]^ However, a significant drawback of current transportation approaches is the limited capacity to produce sufficient antigen‐specific T cell clones, especially CD8^+^ T cells.^[^ ^12^ ^]^ Thus, there still is a demand for an ideal delivery nanocarrier for peptide‐based tumor vaccines.

On the other hand, adjuvants play a critical role in overcoming the low immunogenicity of peptide antigens in vaccines. They are traditionally immunopotentiators that can increase the magnitude of adaptive responses to vaccines or guide the type of immune responses to generate effective forms of immunity, thereby enhancing the efficacy of vaccines.^[^ ^13^ ^]^ For decades, only a handful of adjuvants have been approved for clinical use, such as Alum, MF59, AS01, AS03, AS04, and Cytosine phosphor‐guanosine (CpG) 1018.^[^ ^14^ ^]^ Although the immunostimulatory effects of these adjuvants are thought to be related to the proinflammatory responses downstream of Toll‐like receptors, the systemic inflammation associated with their use is a potential concern for side effects. Therefore, expanding knowledge about adjuvants is crucial to catalyze the development of novel formulations of tumor peptide vaccines.

**Scheme 1:**
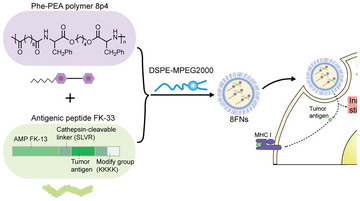
Schematic illustration of the vaccine design, tumor antigen presentation, and innate stimulation in dendritic cells by 8FNs nanovaccine.

LL‐37 (LLGDFFRKSKEKIGKEFKRIVQRIKDFLRNLVPRTES) is a human antimicrobial peptide (AMP) that plays an important role in directly killing the microbial pathogens, modulating DCs maturation, and promoting T cells activation.^[^ ^15^ ^]^ Based on these characteristics, LL‐37 has been reported to act as an immune adjuvant in the treatment of infectious diseases or cancer immunotherapy.^[^ ^16^ ^]^ Structural biological analysis revealed short peptide FK‐13 (FKRIVQRIKDFLR) as the functional core of LL‐37.^[^ ^17^ ^]^ In addition, it has been reported that FK‐13 promoted the phagocytosis of pathogens in monocytes, suggesting that FK‐13 may enhance the antigen uptake process in APCs.^[^ ^18^ ^]^ Therefore, FK‐13 may be a potential adjuvant for peptide‐based tumor vaccines. In this study, we evaluated the adjuvant activity of FK‐13 and found that it strongly promoted the activation of CD8^+^ T cells and DCs. To ensure efficient codelivery of antigen and FK‐13, we synthesized a fusion peptide named FK‐33 by fusing the antigen epitope with FK‐13, and four lysine residues joined by two cathepsin‐degradable linkers. We further used 8p4, an l‐phenylalanine polymer with good biodegradable and biocompatible capacity for FK‐33 peptide formulation and delivery. As our previous work has shown, 8p4 can self‐assemble into nanoparticles for docetaxel loading, and escape from endo/lysosomes to enhance drug delivery and cancer treatment in vivo.^[^ ^19^ ^]^ By formulating FK‐33 with 8p4 nanoparticles, we developed a facile and efficient approach to produce a novel peptide‐based tumor nanovaccine. Using MHC‐I epitopes from two model antigens, ovalbumin (OVA) and tyrosinase‐related protein 2 (Trp2), we demonstrated that this nanovaccine formulation could promote the activation of DCs and trigger a robust antigen‐specific CD8^+^ T cell response (**Scheme** **1**), resulting in significant tumor inhibition. Furthermore, the combination of 8FNs and anti‐PD‐1 treatment significantly suppressed tumor progression and prolonged the survival of tumor‐bearing mice compared to anti‐PD‐1 treatment alone. Overall, this study provides a safe and feasible platform to produce effective nanovaccines for tumor immunotherapy.

## 2 Results

### 2.1 Short Peptide FK‐13 Derived from AMP LL‐37 Has Adjuvant Characteristics

**Figure 1:**
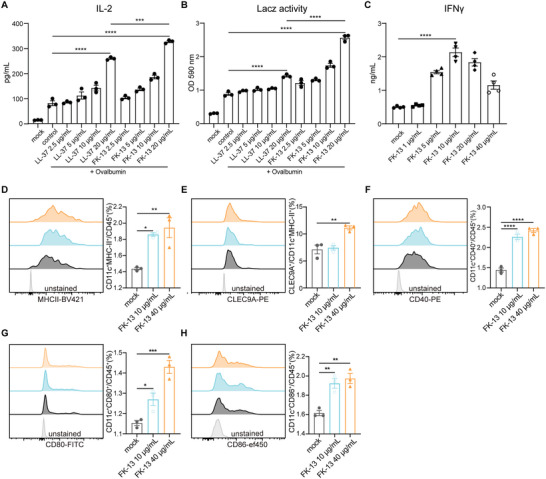
Short peptide FK‐13 derived from AMP LL‐37 has adjuvant characteristics. A,B) BMDCs were treated with LL‐37 or FK‐13 in the presence of OVA (100 µg mL^−1^) for 12 h, followed by coculture with B3Z hybridoma T cells for additional 24 h. Then, B3Z activation was measured by IL‐2 production and LacZ activity. C) CD8^+^ T cells from mouse spleen and lymph nodes were stimulated with FK‐13 ex vivo for 36 h, then IFN*γ* production was measured to reflect CD8^+^ T cell response. D–H) The ratios of CD11c^+^MHC‐II^+^, CD11c^+^MHC‐II^+^CLEC9A^+^, CD11c^+^CD40^+^, CD11c^+^CD80^+^, and CD11c^+^CD86^+^ DCs in the bone marrow cells stimulated with FK‐13 (0, 10, 40 µg mL^−1^) for 72 h. (A–H) Data presented as mean ± SEM. Significance (*P < 0.05, **P < 0.01, ***P < 0.001, ****P < 0.0001) in (A–H) was determined by one‐way ANOVA with Dunnett's multiple comparisons test.

To investigate whether AMP LL‐37 and its core peptide FK‐13 can enhance the antigen presentation process between DCs and T cells, we tested the impact of these peptides on T cell activation according to a previously described antigen presentation protocol.^[^ ^20^ ^]^ Specifically, DCs were primed with antigen together with LL‐37 or FK‐13, then T cells were added, and T cell activation was reflected by IL2 production level and IL2 promoter‐driven *β*‐galactosidase (LacZ) reporter activity. The IL‐2 level and the LacZ activity all increased significantly after LL‐37 and FK‐13 priming in a dose‐dependent manner (**Figure** **1**A,B). We also found that FK‐13 was more effective than LL‐37 in boosting activated T cell‐produced IL‐2 levels at the same concentration. These results suggested that FK‐13 positively impacted the antigen presentation process.

To explore the adjuvant potential of FK‐13, we further examined the effects of FK‐13 on CD8^+^ T cells and DCs individually. IFN*γ* is a cytotoxic cytokine playing critical roles in tumor immunosurveillance and antitumor immune responses.^[^ ^21^ ^]^ Interestingly, we found that FK‐13 promoted IFN*γ* production in murine primary CD8^+^ T cells (Figure 1C), suggesting FK‐13 could directly enhance the activation of CD8^+^ T cells. Conventional DCs (CD45^+^CD11c^+^MHC‐II^+^), especially CLEC9A^+^ type 1 conventional DCs (cDC1) subset, play a critical role in tumor antigens trafficking and antitumor T cell priming, which is closely related to the efficacy of tumor immunotherapies.^[^ ^22^ ^]^ The treatment with FK‐13 promoted the bone marrow cells differentiation into conventional DCs, CLEC9A^+^ cDC1 subpopulation and enhanced the expression levels of costimulatory molecules on DCs, including CD40, CD80, CD86 (Figure 1D–H), indicating the activation of DCs. These results indicate that short peptide FK‐13, derived from AMP LL‐37, could promote antigen presentation and enhance the functions of CD8^+^ T cells and DCs. Based on these findings, we hypothesize that FK‐13 can act as an adjuvant in cancer vaccines and bridge innate immunity and adaptive immunity for a stronger antitumor immune response.

### 2.2 A Novel Tumor Nanovaccine Platform Based on FK‐13 and Phe‐PEA Polymers Has a Potent Capability to Enhance Antigen‐Specific CD8^+^ T Cell Response

The precise delivery of antigens and adjuvants to APCs in vivo through delivery vehicles is critical for specific antitumor immune responses by tumor vaccines.^[^ ^10^ ^]^ To ensure simultaneous delivery of both antigen epitope and peptide FK‐13, we first synthesized a fusion peptide by putting these peptides in the same chain, linked by cathepsin degradable linkers reported in the previous study.^[^ ^23^ ^]^ Specifically, we used the MHC‐I epitope OVA_257‐264_ (SIINFEKL) from OVA as a model antigen. The synthesized whole peptide chain (FK‐33) consisted of 33 amino acids with four lysine residues at the end to increase the hydrophilicity for processing (Figure S1A, Supporting Information). Similar to peptide FK‐13, we found that FK‐33 could upregulate the expression of costimulatory molecules CD40 and CD86 in BMDCs (Figure S1B,C, Supporting Information), indicating that FK‐33 still contained adjuvant properties when carrying antigen peptides.

**Figure 2:**
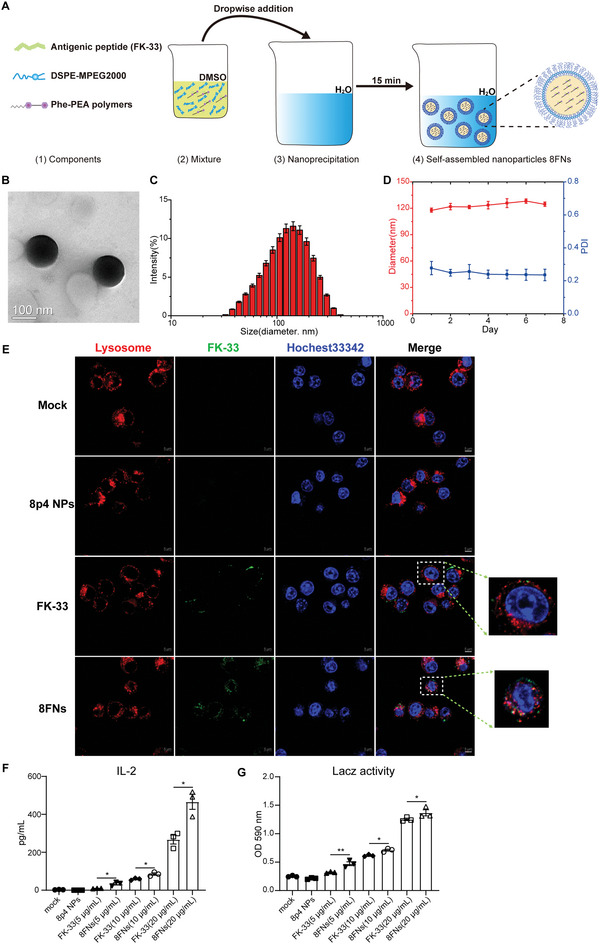
The novel tumor nanovaccine platform based on FK‐13 and Phe‐PEA polymers has a potent activity to enhance antigen‐specific CD8^+^ T response. A) Schematic drawing of 8FNs vaccine formation. Peptide FK‐33 was mixed with 8p4 and DSPE‐MPEG 2000 to form nanoparticles 8FNs by nanoprecipitation. B) The TEM image of 8FNs. Scale bar: 100 nm. C) The average size of 8FNs. D) The size and PDI of 8FNs measured for 7 days. E) The representative images of DC2.4 cells incubated with 8p4 NPs, FITC‐labeled FK‐33 (10 µg mL^−1^) or 8FNs at 6 h by confocal microscopy. The nuclei were stained with Hoechst 33342 (blue), and the endosomes were stained with LysoTracker (red). Scale bar: 5 µm. F,G) BMDCs were treated with peptide FK‐33 or 8FNs for 12 h, followed by coculture with B3Z T cells for an additional 24 h, following which IL‐2 production and LacZ activity were measured. (D,F,G) Data displayed as the mean ± SEM. Significance (*P < 0.05, **P < 0.01) in (F) and (G) was estimated by one‐way ANOVA with Bonferroni's multiple comparisons test.

The encapsulation and delivery of vaccine‐related antigens by nanoparticles have shown promising results in enhancing vaccine efficacy.^[^ ^24^ ^]^ According to previous works, Phe‐PEA polymers can form nanoparticles (NPs) and have the capacity to transport docetaxel, doxorubicin, and small molecule inhibitors like JQ1 and THZ1 to treat cancers.^[^ ^19^, ^25^ ^]^ Thus, we synthesized a mini Phe‐PEA polymer library (8p2, 8p3, 8p4, 8p5, 8p6, and 8p8) with different numbers of methylene (Figure S2A–C, Supporting Information) and further evaluated their potential as the delivery vehicles for FK‐33 nanovaccines. Using the nanoprecipitation strategy,^[^ ^26^ ^]^ we obtained nanoparticles based on FK‐33 and different Phe‐PEA polymers (**Figure** **2**A). The LacZ reporter assay results showed that 8p4 was the best carrier for FK‐33 to induce antigen‐specific CD8^+^ T cell response (Figure S2D,E, Supporting Information), and its chemical structure was confirmed by the H‐NMR spectroscopy (Figure S2F, Supporting Information).

Next, we characterized the physicochemical properties of 8FNs. Transmission electron microscopy (TEM) images showed 8FNs as round‐shaped NPs, with a mean diameter of ≈120 nm (Figure 2B,C). The polydispersity index (PDI) of 8FNs remained at ≈0.25 for one week under 4 °C, and the diameter was also stable (Figure 2D). The average surface potential of 8FNs was near −2 mV (Figure S3A, Supporting Information). Furthermore, we detected the loading efficiency of peptides in 8FNs using fluorescein isothiocyanate (FITC)‐labeled peptide FK‐33 and found that nearly 91% of peptide FK‐33 was loaded by 8p4 (Figure S3B,C, Supporting Information).

Then, we studied the intracellular uptake and trafficking of the 8FNs in DC2.4 cells using confocal laser scanning microscopy. FITC‐labeled FK‐33 as FITC‐8FNs showed that the FITC‐8FNs could efficiently deliver antigenic peptides into DCs with noticeable endo/lysosome escape (Figure 2E). Even after 16 h of incubation, a fraction of nanoparticles could still escape from endo/lysosome (Figure S3D, Supporting Information). The detailed analysis of the subcellular colocalization of 8FNs found that 8FNs partially colocalized with early endosomes (EEA1+), and a small fraction of 8FNs entered late endosomes (Rab7+). 8FNs were rarely found in recycling endosomes (TfR+), which suggested that fewer 8FNs would escape endo/lysosome pathway through exocytosis (Figure S3E, Supporting Information).

We observed a stronger CD8^+^ T cell response in the 8FNs group instead of the FK‐33 group in a dose‐dependent manner, indicating that 8FNs significantly improved the efficiency of antigen delivery in DCs (Figure 2F,G). Compared with FK‐33 or 8p4 NPs, 8FNs most strongly stimulated the activation of DCs (Figure S4A,B, Supporting Information). In addition, the cytokines analysis suggested that 8FNs treatment reduced the production of IL‐10 derived from BMDCs (Figure S4C–G, Supporting Information). We further analyzed the activation status of several important innate immune signaling pathways, including MAPKs, NF‐*κ*B, and STING signaling in BMDCs after 8FNs priming. Except for a moderately increased ERK1/2 and AKT phosphorylation, 8FNs treatment did not strongly activate TBK1, IRF3, p38, JNK1/2, or NF‐*κ*B signaling (Figure S5, Supporting Information).

These findings support the perspective that an effective peptide‐based tumor vaccine, based on the novel adjuvant FK‐13 and Phe‐PEA 8p4 nanoparticles, has been well‐designed. The 8FNs nanovaccine can escape from endo/lysosome and efficiently promote antigen presentation for eliciting specific immune responses.

### 2.3 8FNs Activate DCs in the Lymph Nodes and Elicit Antigen‐Specific CD8^+^ T Cell Response Safely In Vivo

**Figure 3:**
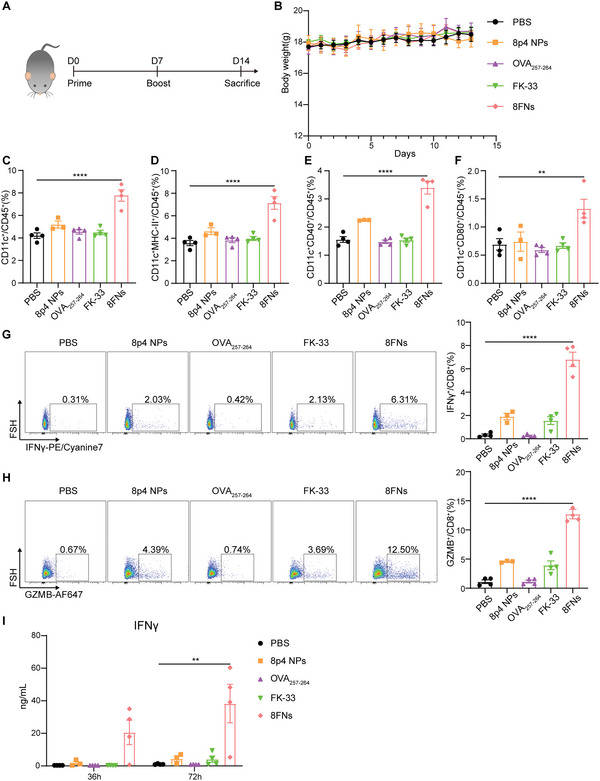
8FNs activate DCs in lymph nodes and elicit specific CD8^+^ T cell response safely in vivo. A) Mice were immunized subcutaneously with PBS (*n* = 4), 8p4 NPs (*n* = 3), peptide OVA_257‐264_ (*n* = 4), peptide FK‐33 (*n* = 4) or 8FNs (*n* = 4) on day 0, day 7 and sacrificed on day 14 for subsequent analysis. B) The mice body weights of the indicated groups for 14 days. C–F) Flow cytometry analysis results of the percentage of CD11c^+^, CD11c^+^MHC‐II^+^, CD11c^+^CD40^+^, and CD11c^+^CD80^+^ DCs in lymph nodes. G,H) Flow cytometry analysis results of the percentage of IFN*γ* ^+^CD8^+^ T cells (G) and the percentage of GZMB^+^CD8^+^ T cells (H) in splenocytes stimulated by peptide OVA_257‐264_ on 72 h. I) The splenocytes were isolated from pretreated mice and stimulated with peptide OVA_257‐264_ (10 µg mL^−1^) in a medium. The IFN*γ* production was measured by ELISA at 36 and 72 h. (B,C–I) Datadisplayed as the mean ± SEM. Significance (**P < 0.01, ****P < 0.0001) in (C–I) was estimated by one‐way ANOVA with Dunnett's multiple comparisons test.

To test the in vivo vaccination effect of 8FNs, we immunized C57BL/6 mice subcutaneously on day 0 and day 7 with (1) PBS, (2) 8p4 NPs, (3) OVA_257‐264_, (4) FK‐33, and (5) 8FNs (**Figure** **3**A). We monitored the body weight of each mouse daily after injection for two weeks and found that the body weights of all group mice were comparable during the treatment course (Figure 3B). Throughout the study, no obvious damage in the heart, liver, lung or kidney was observed on histology (Figure S6A, Supporting Information). On day 14, mice vaccinated with 8FNs had the highest proportion of CD11c^+^ cells and conventional DCs among CD45^+^ cells in lymph nodes (Figure 3C,D), along with increased CD45^+^CD11c^+^CD40^+^ and CD45^+^CD11c^+^CD80^+^ DC subsets (Figure 3E,F). These results indicated that 8FNs significantly promoted the activation of DCs in the lymph nodes. In addition, the immunotyping analysis of lymph nodes also suggested that 8FNs could increase the number of neutrophils instead of natural killer (NK) cells and elicit potent adaptive T cell responses with the remarkable CD44^+^CD62L^−^ effector CD8^+^ T cells (Figure S6B–L, Supporting Information). Next, we investigated the cross‐priming of CD8^+^ T cells after vaccination. Interestingly, when splenocytes were isolated and cultured with peptide OVA_257‐264_ ex vivo, we observed that the percentage of IFN*γ* ^+^ cells in CD8^+^ T cells of the 8FNs group showed a sharp increase (Figure 3G), along with GZMB^+^CD8^+^ T cells (Figure 3H). In line with the flow cytometry results, a robust rise of IFN*γ* secretion was observed in the 8FNs‐vaccinated group (Figure 3I). According to these results, we concluded that 8FNs could induce DC activation in the lymph nodes to efficiently present antigens and elicit strong activation of antigen‐specific CD8^+^ T cell response in vivo.

### 2.4 8FNs Elicit Potent Antitumor Immunity for Tumor Control

**Figure 4:**
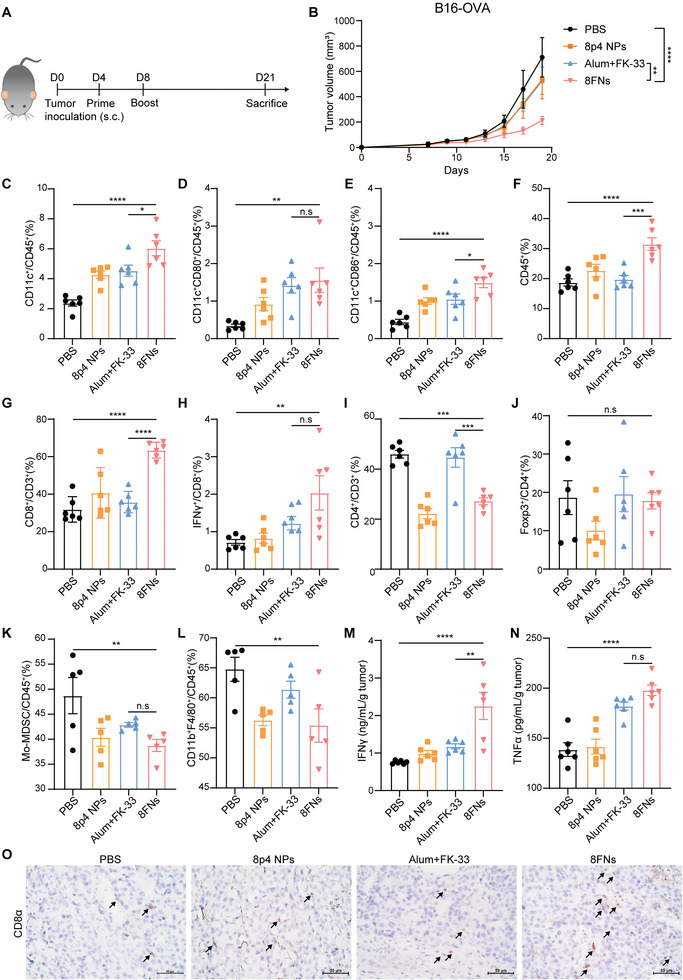
Treatment of 8FNs induces antitumor immune responses in B16‐OVA melanoma. A) Experimental timeline for treatment of B16‐OVA tumor‐bearing mice. C57BL/6 mice were inoculated at the subcutaneous flank with 2 × 10^5^ B16‐OVA melanoma cells on day 0 and vaccinated subcutaneously at the inguinal region of mice on day 4 and day 8 with PBS, 8p4 NPs, Alum+FK‐33, and 8FNs (*n* = 6). s.c., subcutaneous. B) The average tumor growth curves. C–E) Representative flow cytometry analysis results of the percentage of CD45^+^CD11c^+^, CD45^+^CD11c^+^CD80^+^, and CD45^+^CD11c^+^CD86^+^ DCs in lymph nodes. F–L) Flow cytometry analysis results of the percentage of CD45^+^ immune cells, CD8^+^ T cells, IFN*γ* ^+^CD8^+^ T cells, CD4^+^ T cells, Foxp3^+^CD4^+^ T cells, Mo‐MDSCs, and TAMs in tumors. M,N) ELISA analysis results of IFN*γ* and TNF‐*α* in the supernatant of excised tumors from mice. O) Representative CD8*α* staining of the tumors. Scale bar: 50 µm. Black arrows indicate CD8*α* positive staining. (B–N) Data displayed as the mean ± SEM. Significance (n.s, not significance, *P < 0.05, **P < 0.01, ***P < 0.001, ****P < 0.0001) in (B) was estimated by two‐way ANOVA with Tukey's multiple comparisons test, in (C–N) was estimated by one‐way ANOVA with Bonferroni's multiple comparisons test.

To determine whether nanovaccine 8FNs could control tumor growth, we vaccinated B16‐OVA tumor‐bearing mice subcutaneously with PBS, 8p4 NPs, Alum+FK‐33 or 8FNs, respectively, on day 4 and day 8 (**Figure** **4**A). Alum, which contains an aqueous solution of aluminum hydroxide, magnesium hydroxide, and inactive stabilizers, is a well‐studied vaccine adjuvant and was used here as a control.^[^ ^27^ ^]^ The body weight of mice in each group mildly increased during the vaccination (Figure S7A, Supporting Information), indicating the safety of 8FNs treatment. The tumor growth results showed that 8FNs‐treated mice displayed the most significant tumor regression compared to PBS or Alum+FK‐33 group (Figure 4B; Figure S7B–E, Supporting Information). The number of CD11c^+^ DCs in the lymph node was increased after 8FNs treatment, including CD80^+^ and CD86^+^ cells (Figure 4C–E), indicating enhanced DC activation or maturation, which is needed for efficient antitumor immunity by presenting tumor antigens to T cells for tumor recognition and killing.^[^ ^28^ ^]^ The percentages of tumor‐infiltrated CD45^+^ cells, total T cells (CD45^+^CD3^+^), CD8^+^ T cells (CD45^+^CD3^+^CD8^+^), and CD8^+^ effector T cells (CD45^+^CD3^+^CD8^+^IFN*γ* ^+^) were significantly increased in 8FNs‐treated mice (Figure S7F, G, Supporting Information; Figure 4F–H), while CD4^+^ T cells (CD45^+^CD3^+^CD4^+^) were decreased and regulatory T cells (CD45^+^CD3^+^CD4^+^Foxp3^+^) did not show a significant difference (Figure 4I,J). Monocytic myeloid‐derived suppressor cells (Mo‐MDSC) and tumor‐associated macrophages (TAMs) are generally considered immunosuppressive cell populations in the immunosuppressive tumor microenvironment (TME) and contribute to tumor immune evasion.^[^ ^29^ ^]^ In contrast with increased T cells, tumor‐infiltrated Mo‐MDSC (CD45^+^CD11b^+^Ly6C^+^Ly6G^−^) and TAMs (CD45^+^CD11b^+^F4/80^+^) levels were reduced in the 8FNs‐treated group compared to the PBS group (Figure 4K,L). Furthermore, the concentration of cytotoxic cytokines IFN*γ* and TNF*α* in tumor tissues also significantly increased in the 8FNs groups (Figure 4M,N). Immunohistochemical staining of 8FNs‐treated tumor tissues confirmed the increased infiltration of CD8^+^ T cells (Figure 4O). Collectively, these data suggested that 8FNs could reverse the immunosuppressive TME and potentiate anti‐tumor immunity to inhibit tumor growth.

### 2.5 Preventative Vaccination of 8FNs Inhibits Tumor Growth

**Figure 5:**
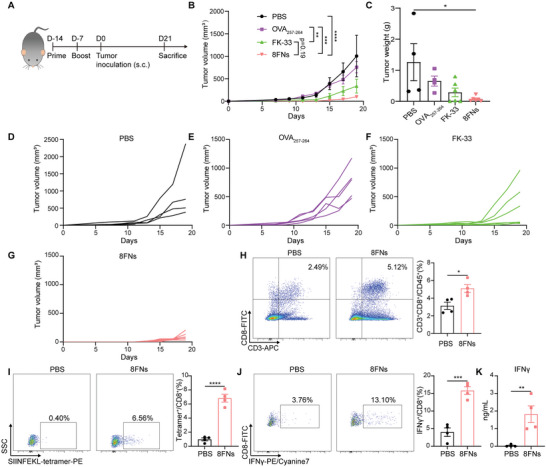
Preventative vaccination of 8FNs inhibits tumor growth. A) Experimental timeline for xenograft animal model. C57BL/6 mice were immunized with PBS, peptide OVA_257‐264_, peptide FK‐33, and 8FNs on days 14 and 7 prior to B16‐OVA tumor inoculation. After receiving two immunization doses, the mice were injected with B16‐OVA tumor cells. Then, two weeks later, the mice were sacrificed for subsequent analysis. PBS, OVA_257‐264_ group (*n* = 4); FK‐33, 8FNs group (*n* = 6). s.c., subcutaneous. B) The growth curves of tumors. C) Tumor weights of the indicated groups measured on day 21 postinoculation. D–G) The growth curved of tumors for the indicated groups. (H,I) Representative flow cytometry dot‐plots and quantification of the frequency of CD8^+^ T cells (H) and SIINFEKL‐specific CD8^+^ T cells (I) in the tumors of PBS and 8FNs group (*n* = 4). J) Splenocytes were isolated from tumor‐bearing mice and stimulated with peptide OVA_257‐264_ (1 µg mL^−1^) in the medium for 72 h ex vivo. Next, flow cytometry analysis was performed to determine the percentage of IFN*γ* ^+^CD8^+^ T cells in splenocytes. K) The concentration of IFN*γ* on the supernatant of splenocytes was measured by ELISA at 72 h. (B,C,H–K) Data displayed as the mean ± SEM. Significance (*P < 0.05, **P < 0.01, ***P < 0.001, ****P < 0.0001) was estimated by two‐way ANOVA with Tukey's multiple comparisons test in (B), one‐way ANOVA with Dunnett's multiple comparisons test in (C), unpaired two‐tailed Student's *t*‐test in (H–K).

We next investigated the antitumor efficacy of 8FNs under the prophylactic treatment setting. C57BL/6 mice were vaccinated 14 and 7 days prior to B16‐OVA tumor inoculation (**Figure** **5**A). We found that tumor growth in 8FNs‐treated mice was significantly slower than that of the control groups, including PBS, OVA_257‐264_, and FK‐33 treated mice (Figure 5B,D–G). Besides, the tumor weight further confirmed that 8FNs treatment efficiently prevented tumor growth (Figure 5C).

Furthermore, we detected the frequency of SIINFEKL‐specific CD8^+^ T cells in the tumors. Based on tetramer staining results, the transplanted tumors in mice treated in advance with nanovaccine 8FNs had a higher percentage of CD8^+^ T cells and antigen‐specific CD8^+^ T cells compared with the PBS groups (Figure 5H,I). Moreover, in the OVA_257‐264_ epitope peptide stimuli setting, splenocytes of 8FNs‐treated mice had a higher proportion of CD8^+^ effector T cells than the PBS group (Figure 5J). Consistent with this finding, splenocytes isolated from vaccinated mice displayed a stronger capacity for IFN*γ* production after being stimulated with OVA_257‐264_ (Figure 5K), indicating a robust antigen‐specific immune response after 8FNs vaccination. The above results suggested that nanovaccine 8FNs effectively triggered antigen‐specific antitumor immune responses and produced a durable immune response for preventing tumor growth.

### 2.6 Nanovaccine 8FNs@Trp2 Inhibits Tumor Metastasis and Enhances the Efficacy of Anti‐PD‐1 Therapy

**Figure 6:**
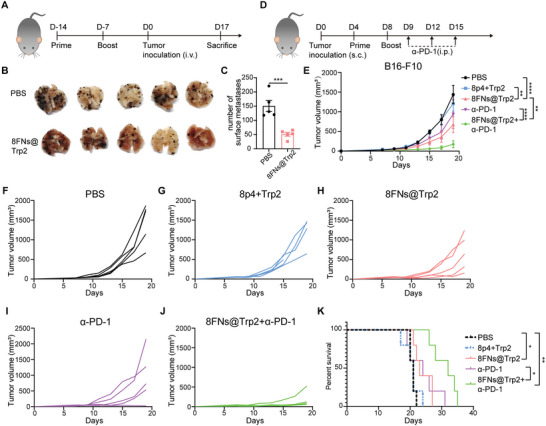
Nanovaccine 8FNs@Trp2 inhibits tumor metastasis and enhances the efficacy of anti‐PD‐1 therapy. A) Experimental timeline for the animal experiment of B16‐F10 metastasis. C57BL/6 mice were immunized with indicated formulations on days 14 and 7 prior to intravenous challenge with B16‐F10 tumor cells, followed by quantification of surface lung colonies (*n* = 5). i.v., intravenous. B,C) The number of surface lung metastases of mice. D) Experimental timeline for treatment of B16‐F10‐bearing mice. C57BL/6 mice were subcutaneously injected with B16‐F10 tumor cells and divided into five groups, then treated with PBS, 8p4+Trp2 nanoparticles, 8FNs@Trp2, *α*‐PD‐1, and 8FNs+*α*‐PD‐1 group. *α*‐PD‐1 antibody was used in a dose of 150 µg per mouse on days 9, 12, and 15, *n* = 5. s.c., subcutaneous; i.p., intraperitoneal. (E–J) The growth curves of tumors. K) The survival curves of indicated groups as described in (D). (C,E) Data displayed as the mean ± SEM. Significance in (C) (***P < 0.001) was estimated by unpaired two‐tailed Student's *t*‐test, in (E) (**P < 0.01, ****P < 0.0001) was estimated by two‐way ANOVA analysis with Tukey's multiple comparisons test, in (K) (*P < 0.05, **P < 0.01) was estimated by log‐rank (Mantel‐Cox) test.

To extend the feasibility of our nanovaccine platform, we tested whether other tumor antigens could also be delivered by the 8FNs platform. Peptide TRP2_181‐188_ (VYDFFVWL) is one of the most widely tested tumor‐associated antigens for murine melanoma B16‐F10 cells.^[^ ^30^ ^]^ Thus, we synthesized a novel antigenic peptide FK‐33@Trp2 based on FK‐13 and TRP2_181‐188_ (Figure S8A, Supporting Information) and prepared the nanovaccine 8FNs@Trp2 with a mean diameter of about 130 nm (Figure S8B, Supporting Information). Consistent with nanovaccine 8FNs, 8FNs@Trp2 also significantly promoted the activation of DCs with the upregulation of costimulatory molecule CD86 (Figure S8C, Supporting Information). It is known that more than 90% of cancer‐related mortality is attributable to metastases rather than primary tumors.^[^ ^31^ ^]^ Thus, we further evaluated the ability of 8FN@Trp2 nanovaccine against lung metastasis of melanomas derived from intravenous injection of B16‐F10 cells (**Figure** **6**A). Strikingly, immunization with 8FNs@Trp2 significantly reduced the number of lung metastases of B16‐F10 melanoma, reflecting an effective and durable antitumor immunity after vaccination (Figure 6B,C). This result suggested that 8FNs@Trp2 exhibited a strong capacity to protect against distant metastasis of tumors.

Immune checkpoint blockade antibodies, such as anti‐PD‐1 and anti‐PD‐L1, have exhibited impressive clinical efficacy on many cancer types, but the response rate remains limited and need to be improved.^[^ ^3^ ^]^ To test whether nanovaccine could improve the therapeutic effect of anti‐PD‐1 treatment in melanoma, we first detected the expression of PD‐1 in the previously mentioned B16‐OVA tumor tissues and observed positive staining of PD‐1, indicating a potential for anti‐PD1 treatment (Figure S9, Supporting Information). We then vaccinated B16‐F10 bearing mice subcutaneously with PBS, 8p4+Trp2, or 8FNs, respectively, on day 4 and day 8 (Figure 6D). 8p4+Trp2 was 8p4 loaded with Trp2 peptide as a nanovaccine control to verify the efficacy of 8FNs@Trp2. Then, mice in the PBS or 8FNs group were further treated with anti‐PD‐1. As expected, nanovaccine 8FNs@Trp2 significantly suppressed B16‐F10 tumor growth compared with the PBS and 8p4+Trp2 treatment groups (Figure 6E–H). Strikingly, combination of 8FNs@Trp2 with anti‐PD1 significantly enhanced the tumor control of anti‐PD‐1 treatment alone and prolonged survival of tumor‐bearing mice (Figure 6I–K), suggesting that 8FNs@Trp2 had a synergistic effect when combined with anti‐PD‐1 therapy.

Collectively, these data demonstrate the flexibility of the 8FNs nanovaccine platform in enhancing the immunogenicity of tumor antigen peptides, such as Trp2, to elicit specific antitumor immunity and inhibit tumor growth. Our work showed that the nanovaccine 8FNs robustly potentiated the therapeutic effect of anti‐PD‐1 in preclinical animal tumor models and provided a new potential combination strategy by using 8FNs together with immune checkpoint inhibitors.

## 3 Discussion

Cancer vaccines typically contain selected tumor antigens and adjuvants that activate APCs, especially DCs, to boost the patient's adaptive immunity against specific tumor antigens, inhibit tumor growth, eradicate minimal residual tumor cells, and establish long‐lasting antitumor memory.^[^ ^32^ ^]^ As a promising immunotherapy, personalized tumor vaccines have recently shown encouraging clinical therapeutic efficacy in multiple cancer types, such as melanoma, glioblastoma, and gastrointestinal cancer.^[^ ^33^ ^]^ This study introduced a novel and versatile peptide‐based nanovaccine platform with potent antitumor capacity. Specifically, we identified the cationic peptide FK‐13 as a valuable adjuvant for peptide‐based tumor vaccines that can elicit a CD8^+^ T cell response and induce the maturation of DCs. By combining the Phe‐PEA polymer 8p4 and modified antigen FK‐33, we developed an efficient and effective in vivo delivery system for peptide‐based tumor nanovaccines, called 8FNs. This feasible and convenient approach significantly prevents tumor progression by reshaping the immunosuppressive tumor microenvironment with increased DCs, reduced Mo‐MDSC and TAMs infiltration, and enhanced antigen‐specific CD8^+^ T cell response in vivo. Furthermore, the peptide‐based nanovaccine 8FNs also potentiate the therapeutic efficacy of anti‐PD‐1 treatment, providing a potential combinational strategy to maximize the efficacy of cancer immunotherapy.

As the active core of host defense peptide LL‐37, short peptide FK‐13 contains nearly full antimicrobial and antifungal activities.^[^ ^34^ ^]^ Structural biology analysis revealed that FK‐13 could self‐assemble into a protein fibril with alternating hydrophobic and positively charged zigzagged belts, which possibly underlie interactions with and subsequent disruption of negatively charged bacterial membranes.^[^ ^35^ ^]^ Although there have been reports showing that FK‐13 can promote phagocytosis of complement‐opsonized pathogens in monocytes by binding with the integrin CD11b/CD18 and actin released from necrotized cells to avoid microbial proteolytic degradation, the immunomodulatory functions of FK‐13 have not been fully understood.^[^ ^18^, ^36^ ^]^ LL‐37 has been reported to promote the activation of DCs.^[^ ^16b^ ^]^ In this study, we found that FK‐13 could further improve the antigen presentation of model antigen OVA than LL‐37 and substantially promote the maturation of DCs. Moreover, FK‐13 can significantly promote bone marrow cell differentiation into CD11c^+^MHC‐II^+^ and cDC1 subpopulations and enhance the expression of costimulatory molecules, including CD40, CD80, and CD86. These activated DCs can play a critical role in tumor antigens trafficking and priming specific T cell immunity.^[^ ^22^, ^37^ ^]^ Additionally, we validated that FK‐13 could stimulate IFN*γ* production by activated CD8^+^ T cells. IFN*γ* plays a pivotal role in tumor immunosurveillance and cancer immunotherapy by increasing MHC expression, promoting antigen presentation, directly inhibiting tumor cell proliferation, and augmenting tumor‐infiltrating immune cells’ functions.^[^ ^21^ ^]^ Collectively, these results confirmed that short peptide FK‐13 could activate both innate and adaptive immunity. Unlike nucleotide‐based immune adjuvants such as CpG1018 or R848, FK‐13 is a short peptide that can be conveniently fused with antigenic peptides to promote APC activation and antigen presentation. These characteristics make FK‐13 a promising adjuvant for peptide‐based tumor vaccines.

Delivery vehicles are essential components for tumor vaccines. The widely used delivery materials for tumor vaccines in clinics are water‐in‐oil emulsions, but the therapeutic efficacy of tumor vaccines based on these formulas is still limited.^[^ ^10^ ^]^ Nanodelivery systems offer a unique opportunity to improve tumor vaccines’ efficacy, with features including tissue targeting, prolonged circulation, and preferential uptake by DCs.^[^ ^38^ ^]^ However, a significant challenge for current nanovaccines is elevating their efficacy in generating tumor antigen‐specific CD8^+^ T cells in vivo.^[^ ^12^ ^]^ Nanoparticles developed from hydrophobic amino acid‐based polymers have been studied for nanovaccines to clear bacterial load.^[^ ^39^ ^]^ In this study, we showed that Phe‐PEA polymers, especially 8p4, could spontaneously bind to FK‐33 with very high efficiency and self‐assemble into nanoparticles in an aqueous solution. A previous study has shown that 8p4 could load docetaxel and significantly inhibit the growth of non‐small‐cell lung cancer.^[^ ^19a^ ^]^ Here, we demonstrated that biodegradable nanovaccine 8FNs could safely and efficiently deliver antigens and elicit strong specific CD8^+^ T cell responses in vitro and in vivo. Our results suggest that the poly(ester amide) based on l‐phenylalanine is a highly efficient delivery vehicle for tumor antigen peptides and can serve as a new versatile nanovaccine platform.

Generally, the clinical efficacy of tumor immunotherapy is based on the magnitude and ability of T cells to infiltrate and kill tumors, mainly CD8^+^ T cells.^[^ ^23^ ^]^ Accordingly, the frequency and killing capacity of tumor antigen‐specific CD8^+^ T cells induced in vivo are critical for the antitumor effect of tumor vaccines. Animal experiment results showed that nanovaccine 8FNs had significant therapeutic and prophylactic effects against the tumor. The prime and boost immunization of 8FNs promoted the activation of DCs in the lymph nodes. The tumor antigen‐specific effector CD8^+^ T cells in splenocytes could be restimulated with antigen OVA_257‐264_, suggesting that 8FNs may have a potential long‐term protective effect against tumor occurrence. Higher levels of effector cytokines IFN*γ* and TNF*α* and tumor‐infiltrated CD45^+^ immune cells after 8FNs vaccination indicated that 8FNs better rejuvenated the immunosuppressed microenvironment compared to aluminum salts‐adjuvanted vaccines. Furthermore, 8FNs also significantly induced the frequency of tumor antigen‐specific CD8^+^ T cells and promoted the infiltration and cytotoxic function of CD8^+^ T cells, which finally induced tumor inhibition. Interestingly, the immunostimulating capacity of 8FNs may also attribute to the delivery vehicle, Phe‐PEA polymer 8p4. A previous study showed that a member of the Phe‐PEA polymers family could degrade into l‐phenylalanine to reduce the level of reactive oxygen species (ROS) in MDSCs, thereby inhibiting their immunosuppressive functions in leukemia.^[^ ^25a^ ^]^ Whether 8p4 in 8FNs has a similar effect on MDSCs deserves further study. To explore whether the nanovaccine platform could be applicable to other antigens, we replaced the model antigen OVA_257‐254_ with Trp2 antigen and produced another nanovaccine 8FNs@Trp2. Excitingly, 8FNs@Trp2 potently inhibited the growth of melanoma B16‐F10 and protected against lung metastasis. Meanwhile, 8FNs@Trp2 achieved a synergistic therapeutic effect with anti‐PD‐1 treatment and significantly prolonged the survival of tumor‐bearing mice. These results verified the feasibility of 8FNs for carrying multiple tumor antigens, indicating the enormous clinical potential of 8FNs nanovaccine platform for personalized neoantigen vaccines and combinational immunotherapy with immune checkpoint blockade antibodies.

## 4 Conclusion

In summary, we have identified the potent adjuvant activity of the peptide FK‐13 and demonstrated that the Phe‐PEA polymer 8p4 was a highly efficient carrier for peptide antigens in vitro and in vivo. By combining 8p4 and FK‐33, we have developed an efficient and versatile platform for peptide‐based tumor nanovaccines. This novel self‐assembling vaccine, 8FNs, can reverse the immunosuppressive microenvironment and elicit potent antitumor immunity by strengthening specific CD8^+^ T cell responses against local and metastatic tumors. 8FNs can potentiate the efficacy of anti‐PD‐1 immunotherapy and have promising prospects for personalized neoantigen tumor vaccines.

## 5 Experimental Section

### Mouse Strains and Cell Lines

Six to eight‐week‐old female C57BL/6 mice were purchased from Beijing Vital River Laboratory Animal Technology Co., Ltd. All animal studies were performed with the approval of the Institutional Animal Care and Use Committee of Sun Yat‐sen University Cancer Center (Approval # L102012021004U). B16‐F10 (murine melanoma cell line) was obtained from the American Tissue Type Collection (ATCC). B16‐OVA cells were constructed by stably expressing OVA cDNA on B16‐F10 cells. The murine DC cell line, DC2.4, was kindly provided by Dr. Kenneth Rock, University of Massachusetts Medical School, Worcester, MA, USA. The murine hybridoma T cells, B3Z, were kindly gifted by Dr. Nilabh Shastri, Johns Hopkins University School of Medicine, Baltimore, Maryland, USA. All cell lines were tested as mycoplasma free and maintained with either DMEM or RPMI 1640 (Invitrogen) supplemented with 10% fetal bovine serum (Gibco) in a humidified incubator at 37 °C and 5% CO_2_ circumstance.

### Reagents and Instruments

l‐phenylalanine (P2126), glycol (102466), 1,2‐propanediol (134368), 1,4‐butanediol (493732), 1,5‐pentanediol (76892), 1,6‐hexanediol (240117), 1,8‐Octanediol (O3303), sebacoyl dichloride (236365), toluene‐4‐sulfonic acid monohydrate (T35920), p‐nitrophenol (241326), dimethyl sulfoxide (DMSO, D2650), and chlorophenol red *β*‐d‐galactopyranoside (220588) were purchased from Sigma‐Aldrich. DSPE‐MPEG2000 (147867‐65‐0) was purchased from Macklin. *β*‐mercaptoethanol (21985023) was purchased from Gibco. MagniSort Mouse CD8^+^ T cell Enrichment Kit (8804‐4622‐74), LysoTracker Red DND‐99 (L7528), Imject Alum (77161), ELISA kits for murine IFN*γ* (88‐7314‐22), IL‐2 (88‐7024‐88), TNF*α* (88‐7324‐88), IL‐6 (88‐7064‐88), IL‐10 (88‐7105‐88), and IL‐12 (88‐7121‐88) were obtained from Thermo fisher scientific. Recombinant murine IL‐2 (212‐12) and FLT3L (250‐31L) were purchased from PeproTech. For the cellular uptake experiments, Hoechst 33342 (C1028) was purchased from Beyotime. LL‐37, FK‐13, FK‐33 (FKRIVQRIKDFLRSLVRVYDFFVWLSLVRKKKK), FITC‐FK‐33, FK‐33@Trp2 (FKRIVQRIKDFLRSLVRVYDFFVWLSLVRKKKK), and antigen peptides including epitope of OVA (SIINFEKL), neo‐epitope of B16‐F10 melanoma Trp2 were synthesized by GL Biochem Ltd (≥95% purity by high‐performance liquid chromatography). The mouse CXCL10 ELISA kit was purchased from R&D systems. The following antibodies were used in this study: Brilliant Violet (BV) 421‐anti CD45 (103134), PE/Cyanine7‐anti CD45 (103114), FITC‐anti CD45 (157214), PE‐anti CD11b (101208), FITC‐anti CD11c (117306), APC‐anti CD11c (117310), PE‐anti CD3 (100206), Alexa Fluor (AF) 700‐anti CD3 (100216), FITC‐anti CD8a (100706), FITC‐anti CD80 (104706), BV421‐anti I‐A/I‐E (107632), APC‐anti Ly‐6C (128016), APC/Cyanine7‐anti‐Ly6G (127624), BV605‐anti CD4 (100451), PE‐anti NK1.1 (156504), PE/Cyanine7‐anti CD44, APC‐anti CD62L, FITC‐anti F4/80, Zombie‐NIR (77184), and PE‐anti CLEC9A/CD370 (143503) were purchased from BioLegend. Super Bright 600‐anti CD4 (63‐0042‐82), eFluor 450 (ef450), anti‐CD86 (48‐0862‐82), PE/Cyanine7‐anti IFN*γ* (25‐7311‐82), and APC‐eFluor 780‐anti Ly‐6G (47‐5931‐82) were purchased from eBioscience. AF647‐anti Foxp3 (560401), PE‐anti CD40 (553791), and AF647‐anti Granzyme B (560212) were purchased from BD‐Bioscience. Tetramer‐SIINFEKL‐PE (TS‐5001‐1C) was purchased from MBL. CD8a antibody (98941S), phospho‐NF‐*κ*B p65 (3033S), NF‐*κ*B p65 (8242S), phospho‐IKK*α*/*β* (2697S), IKK*α* (2682S), phospho‐p38 (4511S), p38 (8690S), phospho‐p44/42 MAPK (Erk1/2, 4370S), Erk1/2 (4695S), phospho‐TBK1 (5483S), TBK1 (3504S), phospho‐IRF‐3 (4947S), IRF‐3 (4302S), phospho‐AKT (13038S), AKT (2920S), Rab7 (9367S), and Alexa‐Fluor 555 (4413S) were obtained from Cell Signaling Technology. Anti‐*β*‐actin‐HRP was from Santa Cruz (sc‐47778). Antibodies against Transferrin Receptor (ab214039), and EEA1 (ab2900) were from Abcam. Anti‐PD‐1 for immunohistochemical staining was from Novus Biologicals (NBP1‐77276), and anti‐PD‐1 antibody for the animal experiment was from BioXcell (BE0273). The protein ladder (DB180) was purchased from MIKX. The optical density value was measured by Tecan Spark10M. Hydrodynamic size and zeta potential were measured using Zetasizer NanoZS90 (Malvern Panalytical). Transmission electron microscope images were acquired using JEM‐14000PLUS TEM (JEOL Ltd). Flow cytometry was performed using LSRFortessa X‐20 (BD Bioscience), and the data were analyzed using FlowJo 10.0 software. The immunofluorescence images were obtained from the Confocal Microscope LSM880 (ZEISS).

### Assembly and Characterization of Phe‐PEA Polymers‐Based Nanovaccines

First, The Phe‐PEA polymers were synthesized according to the previous publications.^[^ ^19^ ^]^ Then, Phe‐PEA polymers (20 mg mL^−1^) and synthesized peptide FK‐33 (2 mg mL^−1^) were mixed at equal volumes in a DMSO solution. Subsequently, DSPE‐MPEG2000 (10 mg mL^−1^) dissolved in DMSO was added, followed by vortex mixing. The weight ratio of Phe‐PEA polymers to DSPE‐MPEG2000 was 5:1. Next, one volume of the mixture was slowly dropped into ten volumes of sterile water with vortex mixing to form nanoparticles 8FNs. The nanoparticles formed instantly for at least 15 min holding at room temperature. After assembly, 8FNs were washed three times with sterile water using a 100 kDa Amico Ultra Centrifugal Filter (UFC910008, Millipore) to remove DMSO and free compounds. Finally, 8FNs were concentrated into a suitable volume of sterile PBS and freshly prepared for in vitro and in vivo experiments. The particle size and zeta potential of 8FNs were characterized by dynamic light scattering using a Zetasizer NanoZS90. The morphology and nanosize of 8FNs were observed under the JEM14000PLUS.

### In Vitro Cell Culture

Bone marrow‐derived DCs (BMDCs) were generated by culturing the bone marrow cells isolated from six to eight‐week‐old female C57BL/6 mice. Briefly, the femur and tibia of mice's hind legs were obtained, and a syringe flushed bone marrow cells with PBS. Then, bone marrow cells were moderately treated with ammonium‐chloride‐potassium (ACK) lysis buffer to remove red blood cells and subsequently resuspended with RPMI 1640 medium containing 10% FBS, recombinant FLT3L (100 ng mL^−1^), 1% penicillin–streptomycin, and *β*‐mercaptoethanol (55 µm). The cell culture medium was refreshed every two days. BMDCs were used nine days after culture. The spleen and lymph nodes were harvested from six to eight‐week‐old female C57BL/6 mice and kept in PBS on ice to isolate primary CD8^+^ T cells. Then, the cells were flushed out from the spleen and lymph nodes by a syringe with PBS. The cell suspension was passed through a 40 µm cell strainer to remove large debris. Next, these cells were centrifuged at 300 × *g* for 5 min, then suspended in ACK lysis buffer to lyse red cells. Subsequently, the cells were washed with PBS and centrifuged at 300 × *g* for 5 min. Afterward, the CD8^+^ T cells were sorted according to the manufacturer's instructions. The CD8^+^ T cells were cultured in RPMI 1640 supplemented with 10% FBS, antibiotics, IL‐2 (100 U mL^−1^), and *β*‐mercaptoethanol.

### In Vitro Antigen Presentation Assay

BMDCs were seeded in 96‐well, U‐bottomed culture plate at a density of 5 × 10^4^ cells per well and pretreated with PBS, peptide LL‐37, FK‐13, FK‐33, or 8FNs in indicated experiments. Then, the cells were pulsed with OVA (100 µg mL^−1^) and cultured for 12 h at 37 °C. Subsequently, the cells were cocultured with 1 × 10^5^ B3Z cells for 24 h. The activation of stimulated B3Z cells was evaluated by IL‐2 concentration in the culture supernatant detected by the murine IL‐2 ELISA kit. The process of LacZ activity measurement was introduced in the previous report.^[^ ^20^ ^]^ Briefly, B3Z cells in the U‐bottomed culture plate were lysed by 50 µL per well lysis buffer and freeze‐thawed. Next, 50 µL PBS containing 0.5% bovine serum albumin and 100 µL substrate solution (1 mg mL^−1^ chlorophenol red *β*‐d‐galactopyranoside) dissolved in *β*‐galactosidase buffer were added to each well. The plate was incubated at 37 °C for 4–12 h until the color reached a fit level, followed by a color intensity reading at 590 nm using Tecan Spark10M.

### T Cells Activation and DC Activation Assay

Primary CD8^+^ T cells were seeded in a 96‐well U‐bottomed culture plate at a density of 5 × 10^5^ cells per well and treated with PBS or a range of FK‐13 for 36 h. Then, the CD8^+^ T cell response was determined by IFN*γ* production. The concentration of IFN*γ* in cell supernatant was measured by ELISA kit. For the DC activation assay, bone marrow cells or BMDCs were treated with peptide FK‐13 for 72 h, then stained with PE/Cyanine7‐anti CD45, APC‐anti CD11c/FITC‐anti CD11c, PE‐anti CLEC9A/CD370, BV421‐anti MHC‐II, PE‐anti CD40, FITC‐anti CD80, and ef450‐anti CD86. After staining, the cells were analyzed by flow cytometry.

### Calculation of the Loading Efficiency of Materials for Antigenic Peptide FK‐33

To calculate the loading efficacy of Phe‐PEA polymers for FK‐33, FITC‐labeled FK‐33 (FITC‐FK33) was used in the experiment. During the cleaning step of 8FNs after assembly, the filtrate after the cleaning was also collected. Then, the fluorescence intensity of nanovaccine FITC‐8FNs and the filtrate were read by Tecan Spark10M. The loading efficacy was calculated as Loading efficacy = the fluorescence of FITC‐8FNs/(the fluorescence of FITC‐8FNs and the filtrate) × 100%.

### Cellular Uptake and Lysosomal Escape of Nanovaccine

To monitor the nanovaccine's uptake, FITC‐labeled peptide FK‐33 (2 µg mL^−1^) was used to prepare FITC‐8FNs. DC2.4 cells were first seeded in confocal wells at a density of 1.5 × 10^5^ cells per well and incubated at 37 °C in 5% CO_2_ for 24 h. Then, the cells were incubated with the medium containing FITC‐8FNs for indicated time points. Next, the cells were treated with LysoTracker Red DND‐99 (75 nm) and Hoechst 33342 (5 µg mL^−1^) for 30 min to detect the colocalization of FITC‐8FNs with lysosomes. After staining, the cells were washed three times with prechilled PBS and analyzed by the confocal microscope LSM880 (ZEISS). To further investigate the pathway that DCs take up the nanovaccine, the inhibitors for phagocytosis (cytochalasin D) and micropinocytosis (amiloride) were used. DC2.4 cells were treated with cytochalasin D (5 µm) and amiloride (20 µm) for 2 h. Then, FITC‐8FNs were added into the medium for 1 h. The cells were stained with LysoTracker Red DND‐99 and Hoechst 33342, following analysis by the confocal microscope. Briefly, for the staining of EEA1, Rab7, and Transferrin Receptor, DC 2.4 cells were incubated with FITC‐8FNs for 4 h. Then, the cells were fixed and stained following the recommended protocol of indicated antibodies. The results were analyzed by confocal microscope.

### In Vivo Immunization of Nanovaccine

To evaluate the immune effect of nanovaccine 8FNs, the six‐week‐old female C57BL/6 mice were subcutaneously injected with PBS, 8p4 NPs (containing 1 mg 8p4 polymer), peptide OVA_257‐264_ (23.5 µg), peptide FK‐33 (100 µg), or 8FNs, in the right groin two times per week, individually. Afterward, the inguinal lymph nodes (LNs) and spleens were harvested. The cells isolated from LNs were collected, filtered through a 40 µm strainer, and stained with PE/Cyanine7‐anti CD45, APC‐anti CD11c/FITC‐anti CD11c, BV421‐anti MHC‐II, PE‐anti CD40, and FITC‐anti CD80 for immune response analysis. The splenocytes isolated from spleens were seeded into a 24‐well plate at a density of 1 × 10^7^ mL^−1^ and cultured with the RPMI 1640 medium containing 10% FBS, antibiotics, *β*‐mercaptoethanol (55 µm), and 10 µg mL^−1^ antigen peptide OVA_257‐264_ for 36 and 72 h. Then, the activated T cells were stained with BV421‐anti CD45, PE‐anti CD3, FITC‐anti CD8a, PE/Cyanine7‐anti IFN*γ*, AF647‐aGZMB, and analyzed by flow cytometry. The supernatant was also collected to measure the concentration of IFN*γ* by ELISA kit. For the analysis of neutrophils, NK cells and adaptive T‐cell responses in lymph nodes, FITC‐anti CD45, PE‐anti CD11b, FITC‐anti F4/80, APC/Cyanine7‐anti Ly6G, PE‐anti NK1.1, AF700‐anti CD3, AF700‐anti CD8, BV605‐anti CD4, PE/Cyanine7‐anti CD44, and APC‐anti CD62L were used for staining followed similar protocols.

### The Assessment of Systemic Toxicity by Body Weight and Hematoxylin and Eosin (H&E) Staining

The systemic toxicity of the nanovaccine was also assessed. Briefly, the mice were subcutaneously inoculated with PBS, 8p4 NPs, peptide FK‐33, or 8FNs. The body weight of treated mice was monitored over the next two weeks. Then, the major organs (heart, lung, liver, kidney) of mice were collected and fixed in 4% paraformaldehyde, further stained with H&E, and imaged using a light microscope (ECLIPSE Ni, Nikon).

### Tumor Studies

B16‐OVA cells were injected subcutaneously (s.c.) in the right flank of six‐week‐old female C57BL/6 mice (2 × 10^5^ cells per mouse) to evaluate the ability of nanovaccine to inhibit the growth of established tumors. Then, the mice were randomly divided into four groups. On days 4 and 8 postinjection, mice were administrated 100 µL PBS, 8p4 NPs, Imject Alum+FK‐33, and 8FNs in the right groin, respectively. The usage of antigenic peptide FK‐33 was 100 µg per mouse. Imject Alum is a typical adjuvant containing an aqueous solution of aluminum hydroxide, magnesium hydroxide, and inactive stabilizers that could deliver antigen peptides to cause an immune response.^[^ ^27^ ^]^ Alum was used following the manufacturer's instructions. The tumor length and width were measured every other day via caliper. Tumor volume was calculated using the formula: 0.5 × tumor length × (tumor width)^2^. To evaluate the ability of nanovaccine to prevent the growth of tumors, six‐week‐old female C57BL/6 mice received two doses of PBS, OVA_257‐264_, FK‐33, and 8FNs, respectively. Two weeks later, 2 × 10^5^ B16‐OVA cells were injected s.c. in the right flank of mice. The tumor volume was monitored, and tumor‐bearing mice splenocytes were stimulated with peptide OVA_257‐264_ (1 µg mL^−1^) to analyze specific immune responses. For in vivo therapeutic efficacy of nanovaccine 8FNs@Trp2 and anti‐PD‐1 immunotherapy, 2 × 10^5^ B16‐F10 cells were injected s.c. in C57BL/6 mice. The tumor‐bearing mice were randomly divided into five groups. The groups received the injection of PBS, 8p4+Trp2, 8FNs@Trp2, and one cohort of mice receiving PBS or 8FNs@Trp2 was supplemented with intraperitoneal (i.p.) administration of anti‐PD‐1 (150 µg in 100 µL PBS per mouse) on days 9, 12, and 15. Tumor length and width were measured every other day, and the tumor volume was calculated according to the formula: 0.5 × tumor length × (tumor width)^2^. Animals were euthanized when tumor volume reached 2000 mm^3^. Furthermore, to investigate the ability of nanovaccine 8FNs@Trp2 to prevent lung colonization by tumor cells, six‐week‐old female C57BL/6 mice were immunized as described above. Then, the mice were challenged with 1.6 × 10^5^ B16‐F10 via a caudal vein injection. At 17 days postinjection, the mice were sacrificed, and the lungs were collected and fixed in 4% paraformaldehyde. The visible surface metastases of the lungs were counted.

### Cytokine Assay of Tumor Tissues

B16‐OVA melanoma tumor‐bearing mice were treated as described above. On day 21 post injection, the tumors were collected, weighed, and homogenized with a prechilled lysis buffer containing a protease inhibitor cocktail. The tissue fragments were removed by centrifugation at 12000 rpm for 15 min at 4 °C. IFN*γ* and TNF*α* levels in the supernatant of tumors were measured by ELISA kits following the manufacturer's instructions.

### Analysis of Tumor‐Infiltrating and Lymph Nodes Leukocytes

The B16‐OVA tumor models were established in tumor studies. The tumors were harvested on day 21, and single‐cell suspensions were generated by a freezing grinding mill (70 Hz, 10 s, LUKA, Guangzhou). The cells were then incubated with the following antibodies to distinguish immune cells populations: BV421‐anti CD45 for leukocytes; APC‐anti CD3, FITC‐anti CD8, PE/Cyanine7‐anti IFN*γ* for CD8^+^ T cells; AF700‐anti CD3, Super Bright 600‐anti CD4, AF647‐anti Foxp3 for CD4^+^ T cells; PE‐CD11b, FITC‐F4/80, APC‐anti Ly‐6C, APC‐eFluor 780‐anti Ly‐6G for macrophages and Mo‐MDSC. The analysis of DCs in lymph nodes has been described in the study of in vivo immunization of nanovaccine.

### Immunohistochemical Analysis

The establishment and harvest of B16‐OVA tumors have been mentioned above. Then, the tumors were fixed in a 4% paraformaldehyde solution, embedded in paraffin, sectioned, and processed for CD8*α*, PD‐1, and PD‐L1 staining. A light microscope observed these stained sections in at least three random fields of view.

### Statistical Analysis

Statistical analyses were performed with the GraphPad Prism software, V.8.0. A two‐tailed unpaired Student's *t*‐test was used to compare two groups, and one‐way of variance (ANOVA) or two‐way ANOVA with multiple comparisons test to compare multiple groups. Survival was estimated, and the comparison of survival curves was calculated by the log‐rank (Mantel‐Cox) test in the GraphPad software. All data are presented as means ± SEM. A *P*‐value of <0.05 was used for determining statistical significance.

## Conflict of Interest

The authors declare no conflict of interest.

## Author Contributions

C.X. and X.Y. contributed equally to this work. J.W. and X.X. conceived and designed the study. C.X. and X.Y. conducted most experiments and analyzed data. C.X. drafted the manuscript. L.W. and T.T. contributed to nanovaccine. H.Z., J.L., Y.L., Z.N., R.Y., M.L., X.W., L.C., H.Z., H.G., and C.L. performed some cell and animal experiments. J.W. and X.X. supervised the project and contributed in writing and revising the manuscript.

## Supporting information

Supporting Information

## Data Availability

The data that support the findings of this study are available from the corresponding author upon reasonable request.
