# Supplementary material for: A Nanovaccine Based on Adjuvant Peptide FK‐13 and l‐Phenylalanine Poly(ester amide) Enhances CD8+ T Cell‐Mediated Antitumor Immunity
Source: Adv Sci (Weinh). 2023 May 10;10(20):2300418. doi: 10.1002/advs.202300418 (PMC10369282; doi:10.1002/advs.202300418)
Supplement: Supplementary file 1 — Supporting Information [file ADVS-10-2300418-s001.pdf]

ADVANCED SCIENCE
Open Access

Supporting Information

for *Adv. Sci.*, DOI 10.1002/advs.202300418

A Nanovaccine Based on Adjuvant Peptide FK-13 and L-Phenylalanine Poly(ester amide) Enhances $CD8^{+}$ T Cell-Mediated Antitumor Immunity

Chunyuan Xie, Xinru You, Hongxia Zhang, Jiahui Li, Liying Wang, Yongxiang Liu, Zining Wang, Ruhui Yao, Tong Tong, Mengyun Li, Xiaojuan Wang, Lei Cui, Huanling Zhang, Hui Guo, Chunwei Li, Jun Wu and Xiaojun Xia**

((Supporting Information can be included here using this template))

Supporting Information

A nanovaccine based on adjuvant peptide FK-13 and L-phenylalanine poly(ester amide) enhances $CD8^+$ T cell-mediated antitumor immunity

Chunyuan Xie, Xinru You, Hongxia Zhang, Jiahui Li, Liying Wang, Yongxiang Liu, Zining Wang, Ruhui Yao, Tong Tong, Mengyun Li, Xiaojuan Wang, Lei Cui, Huanling Zhang, Hui Guo, Chunwei Li, Jun Wu, Xiaojun Xia**

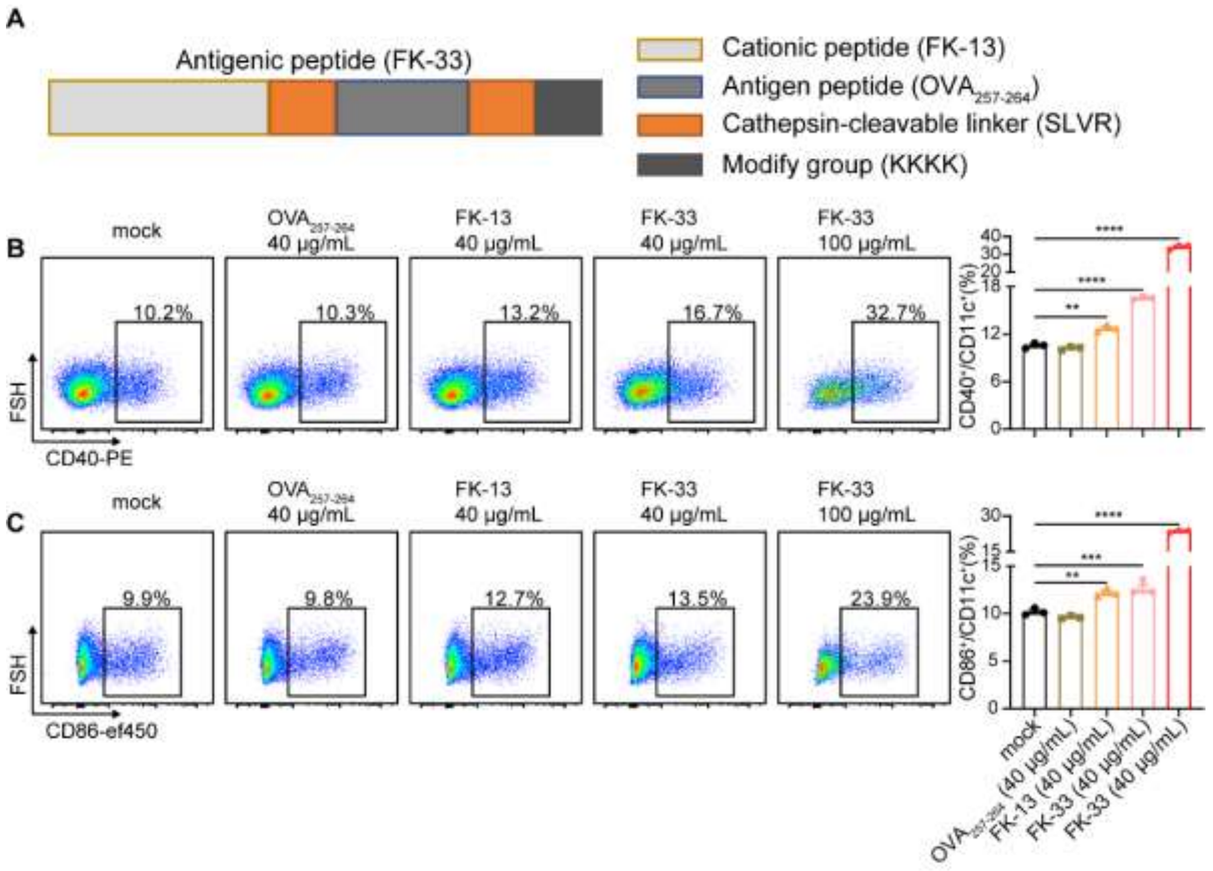

Figure S1. Synthesis and functional characterization of antigenic peptide FK-33.

(**A**) Schematic design of the antigenic peptide FK-33.

(**B**) The ratio of $CD45^+CD11c^+CD40^+$ BMDCs after being treated with mock, $OVA_{257\text{-}264}$, FK-13 or FK-33 for 48 hours (n = 3).

(**C**) The ratio of $CD45^+CD11c^+CD86^+$ BMDCs after being treated with mock, $OVA_{257\text{-}264}$, FK-13 or FK-33 for 48 hours (n =3).

Data in (**B**, **C**) are presented as mean ± SEM. Significance in (**B**, **C**) (***P* < 0.01, ****P* < 0.001, *****P* < 0.0001) was estimated by one-way ANOVA with Dunnett’s multiple comparisons test.

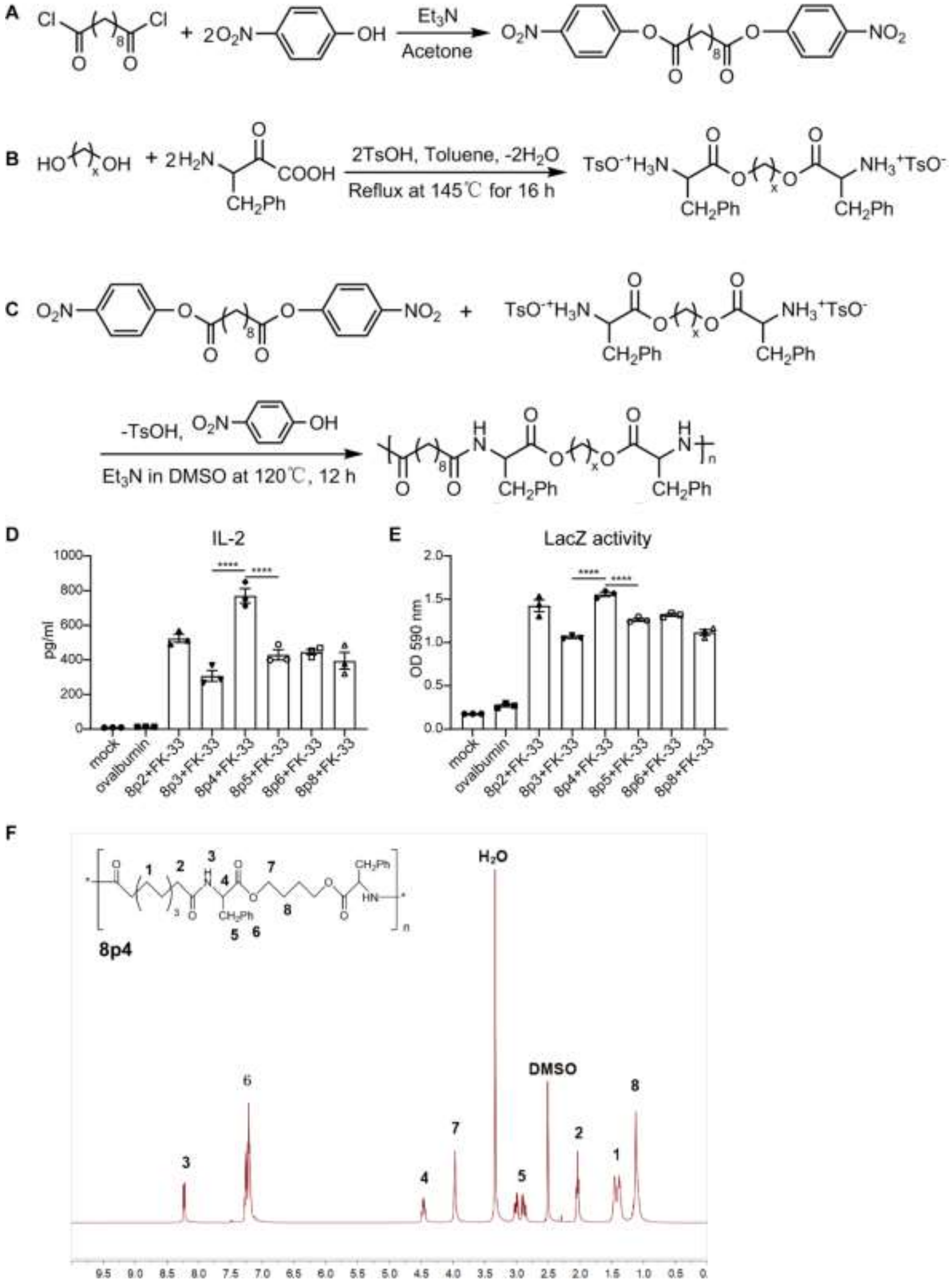

Figure S2. Screening of suitable Phe-PEA polymers to load antigenic peptide FK-33.

(**A-C**) Synthesis of Phe-PEA, x presents the numbers of methylene in diol.

(**D**, **E**) BMDCs were treated with nanoparticles based on Phe-PEA polymer and FK-33 for 12 hours, followed by co-culture with B3Z hybridoma T cells for additional 24 hours, then B3Z activation was measured by IL-2 production and LacZ activity.
(**F**) H-NMR spectra for 8p4 polymers.
Data in (**D**, **E**) are presented as mean ± SEM. Significance in (**D**, **E**) (****$P < 0.0001$) was estimated by one-way ANOVA with Tukey's multiple comparisons test.

A

Sample	Zeta potential (mV)
1	-1.81
2	-2.23
3	-1.97
Mean ± SD	-2.00 ± 0.17

B

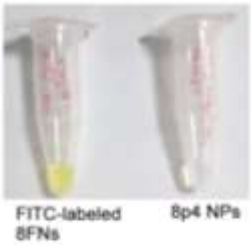

C

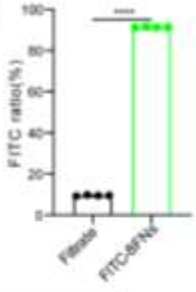

D

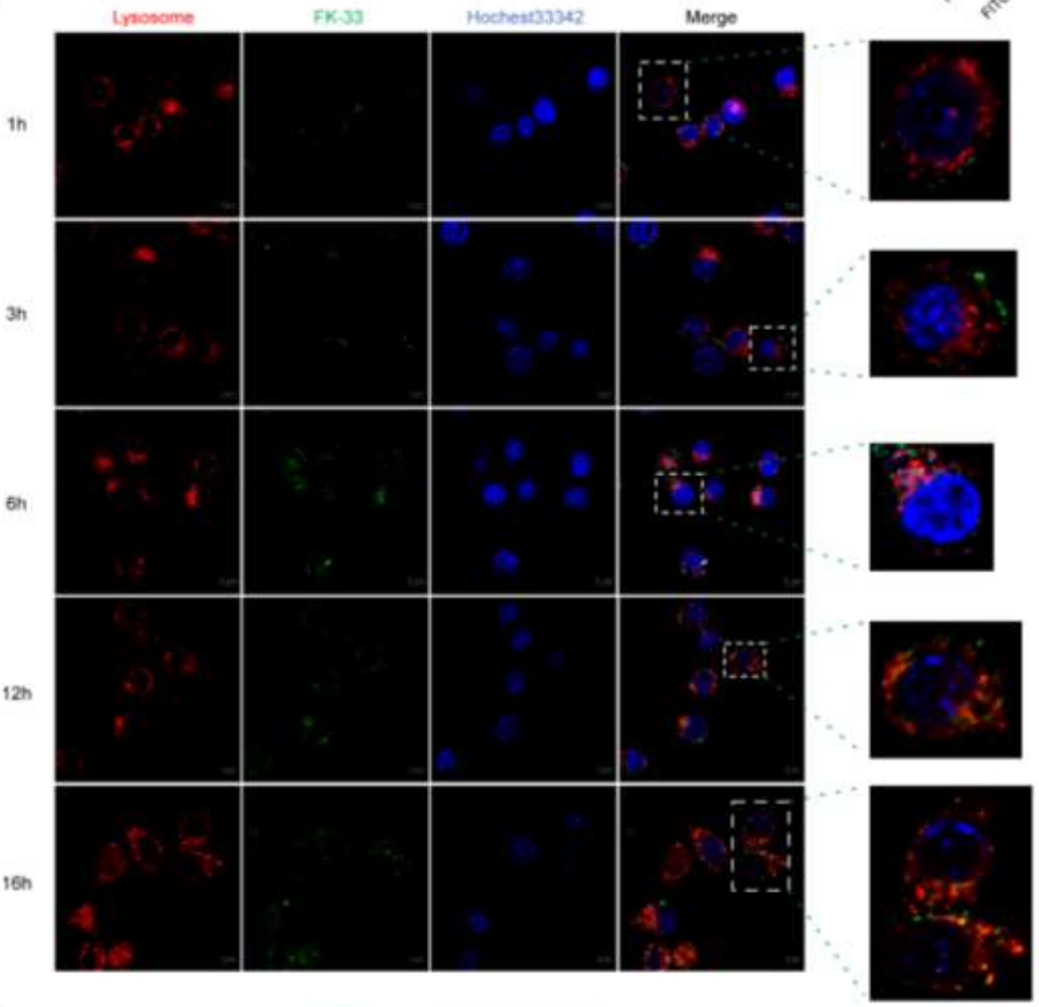

E

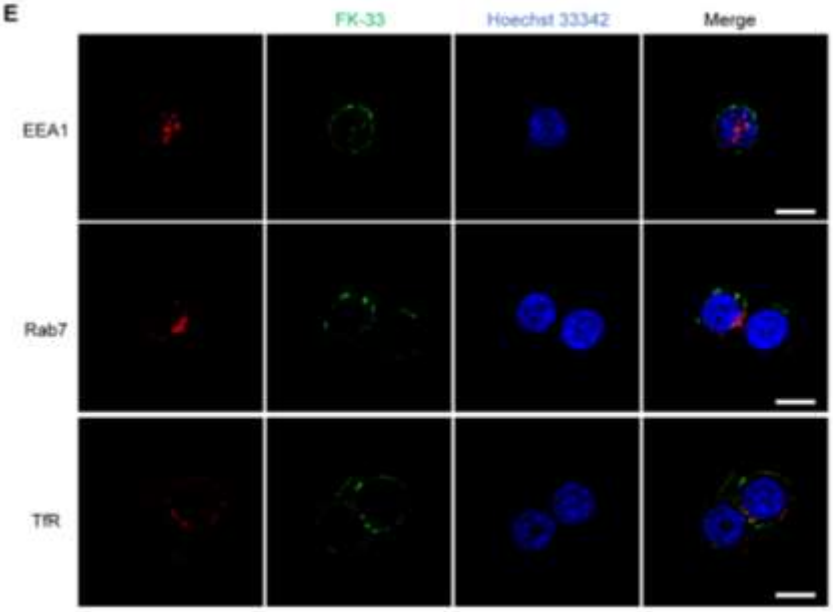

Figure S3. The characteristics and cellular uptake of nanovaccine 8FNs.

(**A**) The Zeta potential of 8FNs.

(**B**) Brightfield image of the FITC-labeled 8FNs and the material control, 8p4 NPs.

(**C**) The loading efficiency of peptides in 8FNs by using FITC-labeled FK-33.

(**D**) Cellular uptake of 8FNs (green) in DC2.4 cells at different time points was characterized by confocal microscopy. 8FNs were able to partially escape from endo/lysosomes. The nuclei were stained with Hoechst 33342 (blue) and the endosomes were stained with LysoTracker (red). Scale bar, 5 μm.

(**E**) Representative subcellular co-localization of 8FNs (green) with different endosomal compartments (red) in DC2.4 cells at 4 hours after co-incubation. TfR, transferrin receptor. The nuclei were stained with Hoechst 33342 (blue). Scale bar, 10 μm.

Data in (**C**) are presented as mean ± SEM. Significance (****P < 0.0001) was estimated by unpaired two-tailed student's t-test in (**C**).

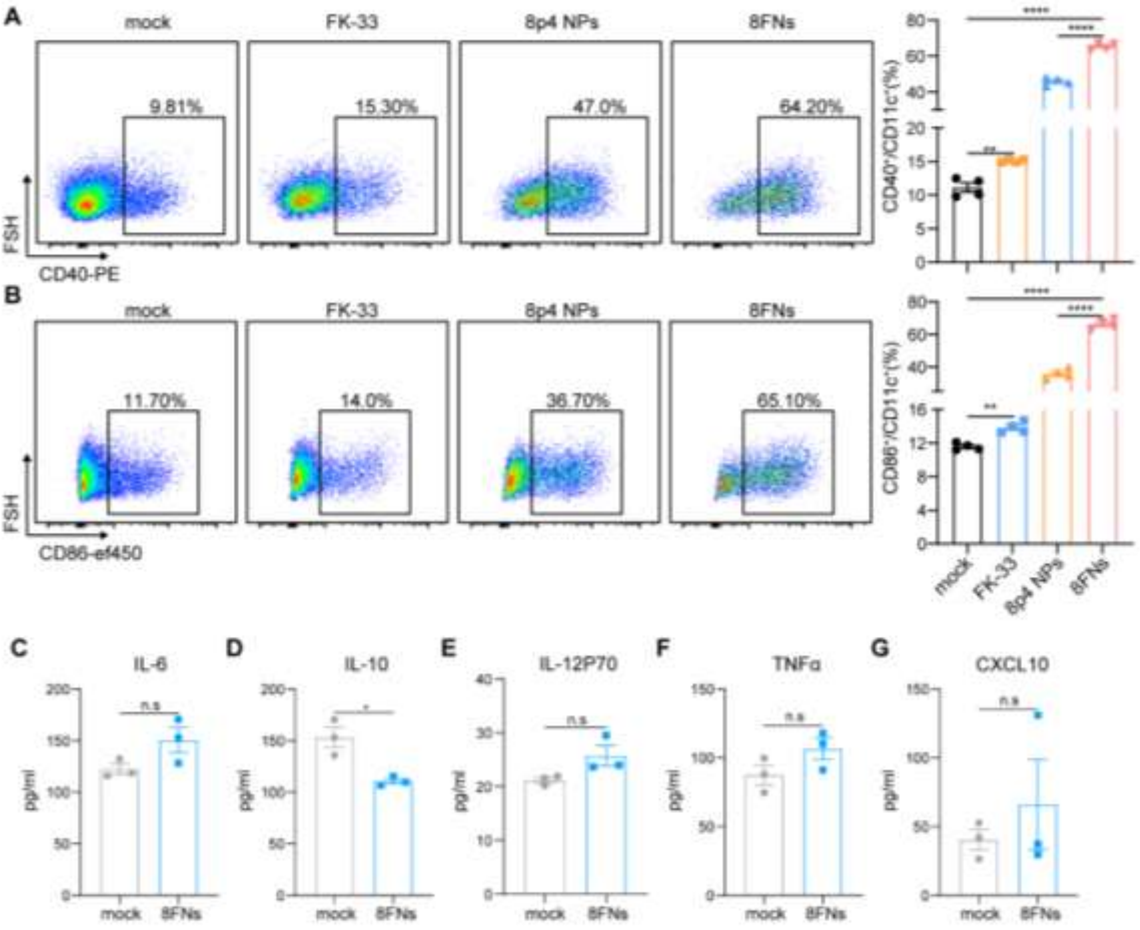

Figure S4. 8FNs promote the activation and cytokine release of BMDCs.

(**A**) The ratio of CD45$^+$CD11c$^+$CD40$^+$ BMDCs after being treated with mock, FK-33 (40 μg/mL), 8p4 NPs, or 8FNs for 48 hours (n = 4).

(**B**) The ratio of CD45$^+$CD11c$^+$CD86$^+$ BMDCs after being treated with mock, FK-33, 8p4 NPs, or 8FNs for 48 hours (n = 4).

(**C-G**) The concentration of IL-6, IL-10, IL-12P70, TNFα, and CXCL10 in the supernatant of BMDCs measured by ELISA after 24 hours treatment (n = 3).

Data in (**A**-G) are presented as mean ± SEM. Significance (n.s, not significance, *P < 0.05, **P < 0.01, ****P < 0.0001) was estimated by one-way ANOVA with Bonferroni's multiple comparisons test in (**A, B**), unpaired two-tailed student's t-test in (**C-G**).

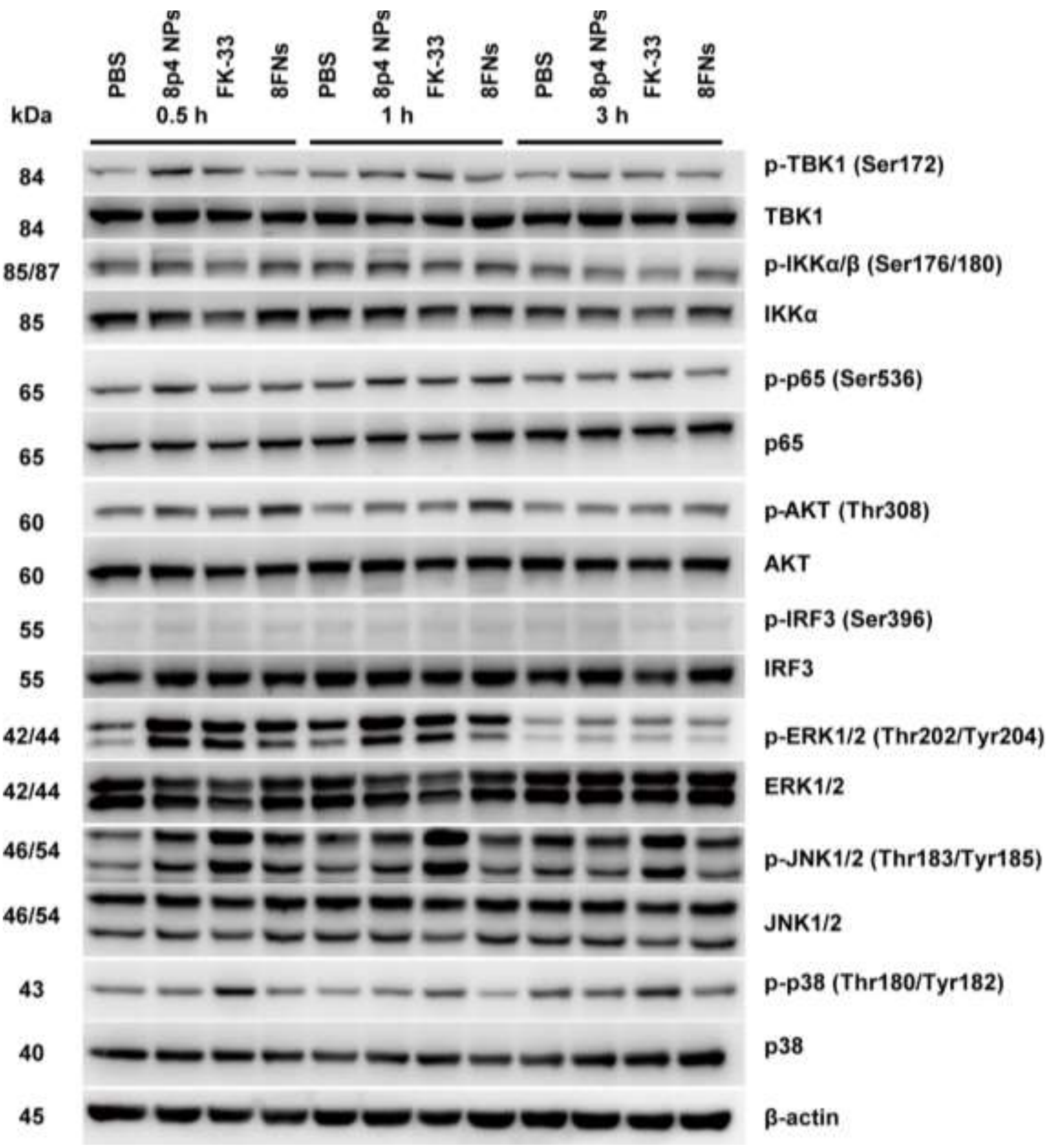

Figure S5. Innate immune signaling pathway activities in BMDCs after different treatments at indicated time points.

BMDCs were treated with mock, FK-33 (40 μg/mL), 8p4 NPs, or 8FNs, and the protein was harvested at indicated time points. Then, the total and phosphorylation levels of innate immune signaling pathway proteins was analyzed by Western blot.

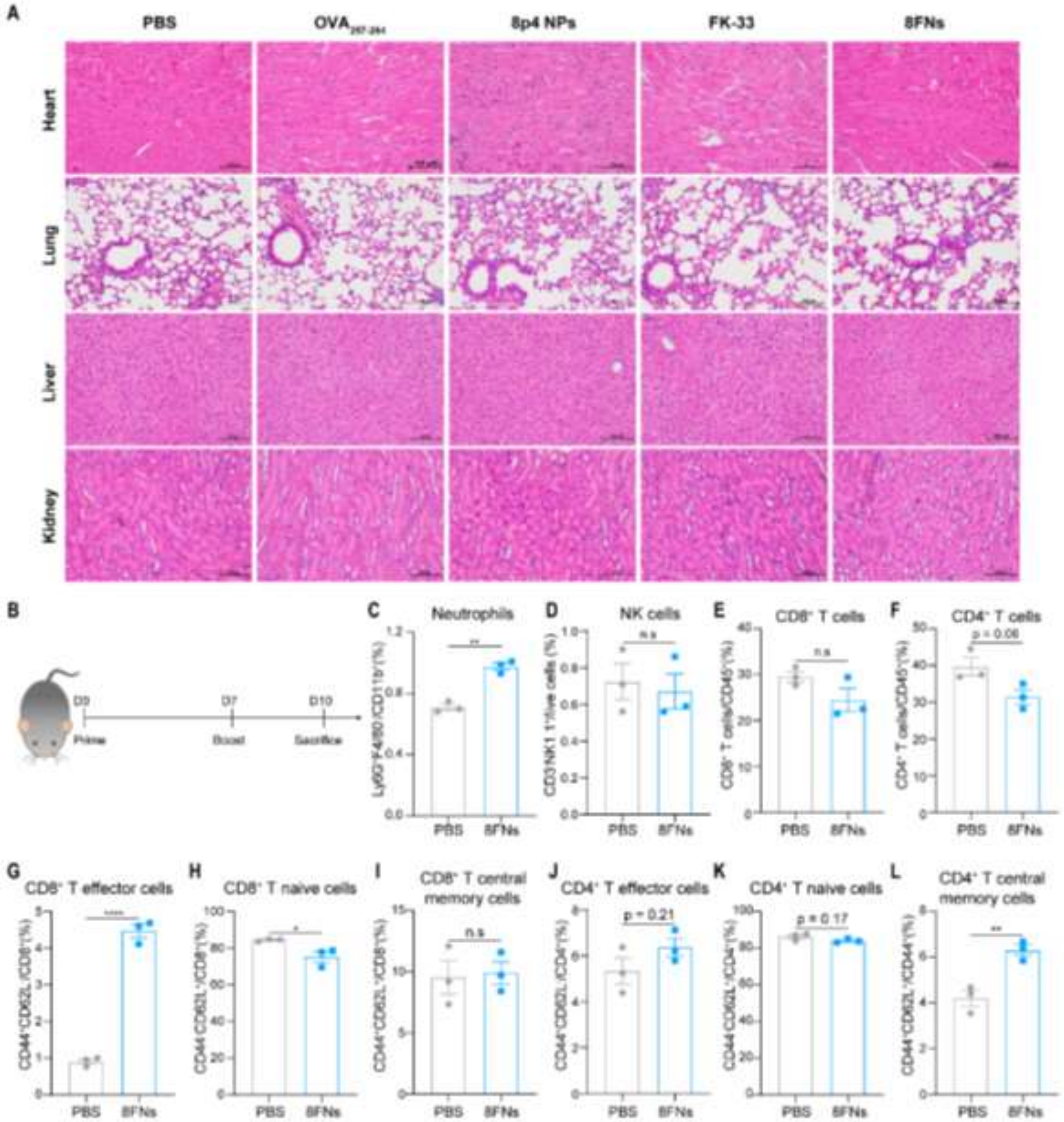

Figure S6. The histological staining of major organs and immune cells analysis in lymph nodes of immunized mice.

(**A**) Representative image of H&E staining of heart, lung, liver, and kidney of immunized mice. Scale bar, 100 μm.

(**B**) Mice were immunized subcutaneously with PBS (n =3) or 8FNs (n =3) on day 0 and day 7, and then sacrificed on day 10 for subsequent analysis.

(**C-L**) Flow cytometry analysis results of the percentage of neutrophils ($CD11b^+F4/80^-Ly6G^+$), NK cells ($CD3^-NK1.1^+$), $CD8^+$ T cells ($CD45^+CD4^-CD8^+$), $CD4^+$ T cells ($CD45^+CD4^+CD8^-$), effector T cells ($CD44^+CD62L^-$), naïve cells ($CD44^-CD62L^+$), and central memory cells ($CD44^+CD62L^+$) in lymph nodes.

Data in (**C-L**) are presented as mean ± SEM. Significance (n.s, not significance, **P* < 0.05, ***P* < 0.01, *****P* < 0.0001) was estimated by unpaired two-tailed student's t-test in (**C-L**).

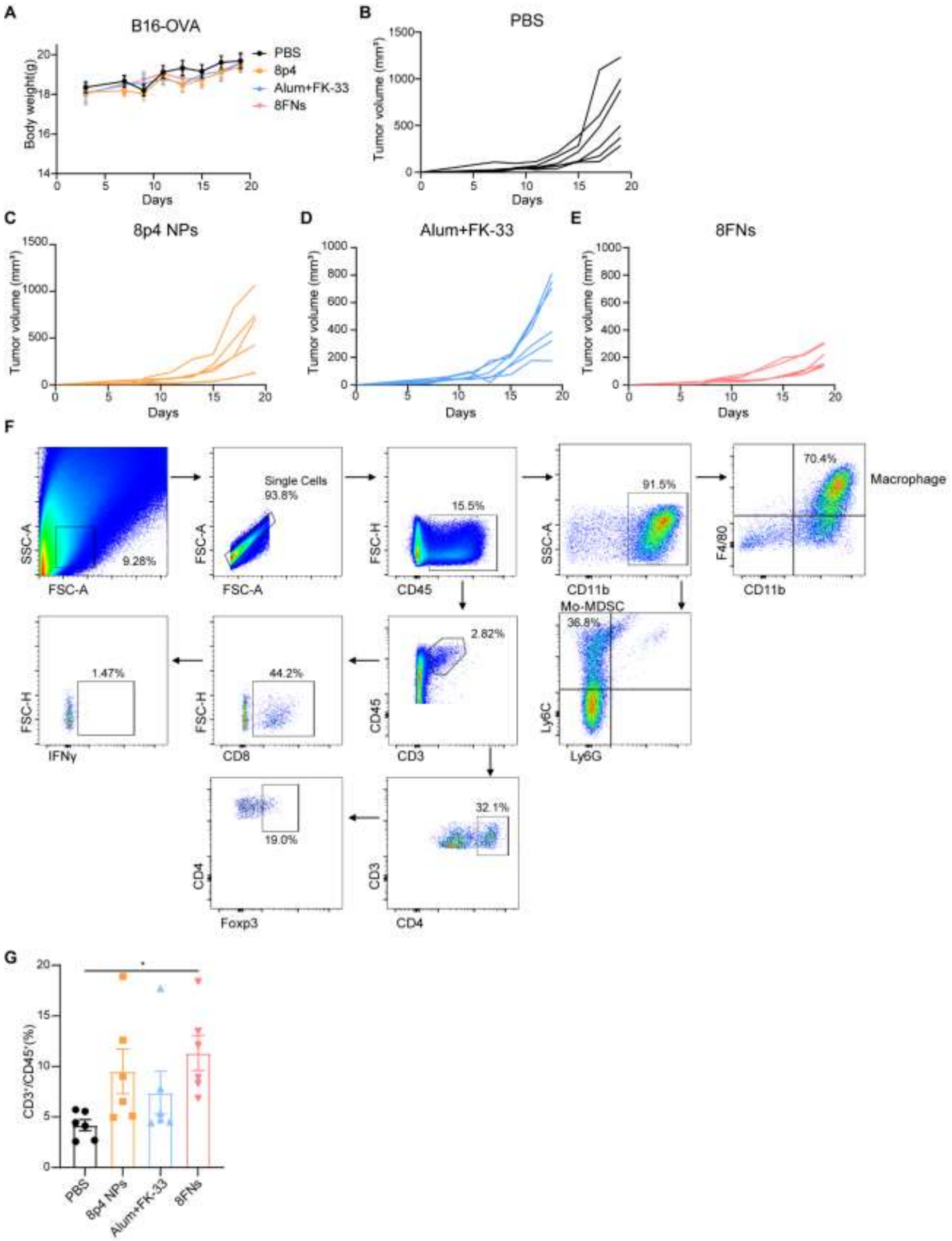

Figure S7. 8FNs effectively inhibit tumor growth and stimulate tumor-infiltrating immune cells.

(**A**) The average body weights of tumor-bearing mice after immunization (n = 6).

(**B-E**) The individual tumor growth curves of different groups (n = 6).
(**F**) Schematic diagram of flow cytometry analysis of tumor-infiltrated immune cells.
(**G**) The percentage of tumor-infiltrated T cells ($CD45^+CD3^+$) in $CD45^+$ cells in different groups (n = 6).
Data in (**A**, **G**) are presented as mean ± SEM. Significance (**P* < 0.05) in (**G**) was estimated by one-way ANOVA with Dunnett's multiple comparisons test.

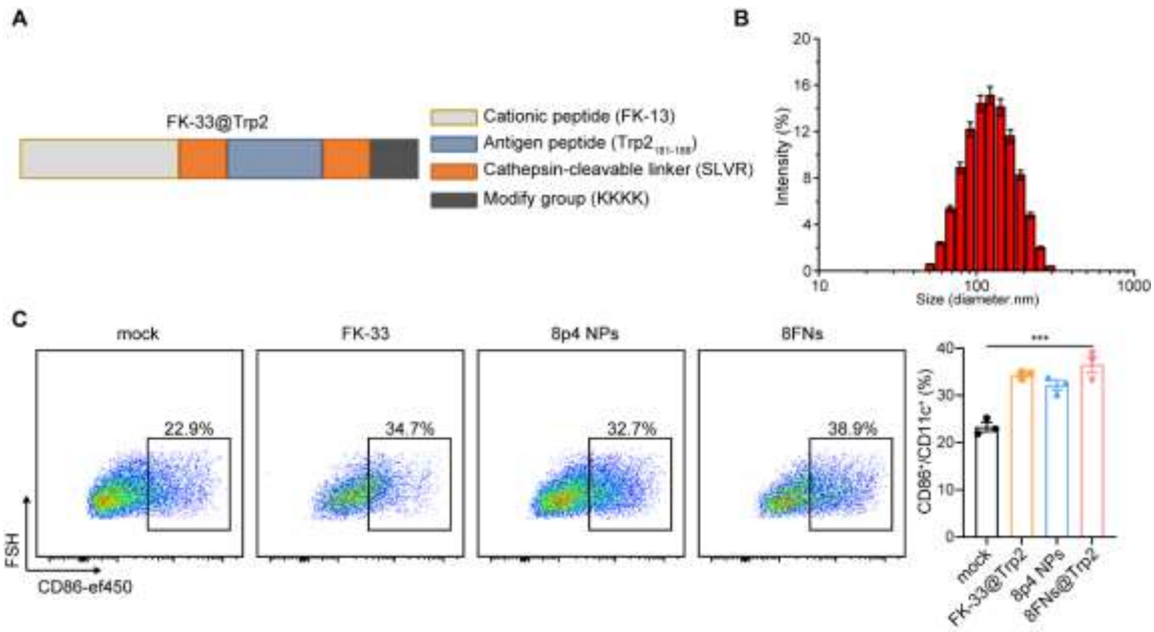

Figure S8. Preparation and the characterization of nanovaccine 8FNs@Trp2.
(**A**) Schematic design of antigenic peptide FK-33@Trp2.
(**B)** The average size of 8FNs@Trp2.
(C) The ratio of $CD45^+CD11c^+CD86^+$ BMDCs after being treated with mock, FK-33@Trp2 (40 μg/mL), 8p4 NPs, or 8FNs@Trp2 for 48 hours (n = 4).
Data in (**C**) are presented as mean ± SEM. Significance (*****P* < 0.0001) was estimated by one-way ANOVA with Dunnett's multiple comparisons test.

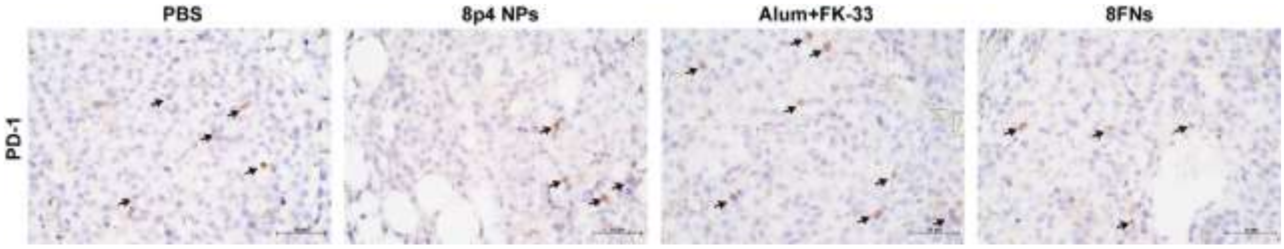

Figure S9. Representative immunohistochemical staining images for PD-1 expression in B16-OVA melanoma tissues.
Scar bar, 50 μm. Black arrows indicate positive PD-1 staining.
